# Dynamic occupancy modeling of temperate marine fish in area‐based closures

**DOI:** 10.1002/ece3.4493

**Published:** 2018-09-21

**Authors:** Jay Calvert, Chris McGonigle, Suresh Andrew Sethi, Bradley Harris, Rory Quinn, Jon Grabowski

**Affiliations:** ^1^ School of Geography & Environmental Science University of Ulster Coleraine UK; ^2^ U.S. Geological Survey New York Cooperative Fish and Wildlife Research Unit Cornell University Ithaca New York; ^3^ Fisheries, Aquatic Science and Technology Laboratory Alaska Pacific University Anchorage Alaska; ^4^ Marine Science Center Northeastern University Nahant Massachusetts

**Keywords:** detectability, dynamic occupancy model, hydrodynamics, temperate marine fish

## Abstract

Species distribution models (SDMs) are commonly used to model the spatial structure of species in the marine environment, however, most fail to account for detectability of the target species. This can result in underestimates of occupancy, where nondetection is conflated with absence. The site occupancy model (SOM) overcomes this failure by treating occupancy as a latent variable of the model and incorporates a detection submodel to account for variability in detection rates. These have rarely been applied in the context of marine fish and never for the multiseason dynamic occupancy model (DOM). In this study, a DOM is developed for a designated species of concern, cusk (*Brosme brosme*), over a four‐season period. Making novel use of a high‐resolution 3‐dimensional hydrodynamic model, detectability of cusk is considered as a function of current speed and algae cover. Algal cover on the seabed is measured from video surveys to divide the study area into two distinct regions: those with canopy forming species of algae and those without (henceforth bottom types). Modeled estimates of the proportion of sites occupied in each season are 0.88, 0.45, 0.74, and 0.83. These are significantly greater than the proportion of occupied sites measured from underwater video observations which are 0.57, 0.28, 0.43, and 0.57. Individual fish are detected more frequently with increasing current speed in areas lacking canopy and less frequently with increasing current speed in areas with canopy. The results indicate that, where possible, SDM studies for all marine species should take account of detectability to avoid underestimating the proportion of sites occupied at a given study area. Sampling closed areas or areas of conservation often requires the use of nonphysical, low impact sampling methods like camera surveys. These methods inherently result in detection probabilities less than one, an issue compounded by time‐varying features of the environment that are rarely accounted for marine studies. This work highlights the use of modeled hydrodynamics as a tool to correct some of this imbalance.

## 1 INTRODUCTION

The use of species distribution modeling as a tool for scientists and environmental managers has seen a substantial increase in the last three decades, driven by a growing demand for knowledge of species’ ranges and facilitated by increasingly powerful computing resources (Barbosa & Schneck, 2015). Species distribution models (SDMs) mathematically represent the relationship between species records and features of the environment, often with the intention of predicting suitable ranges for the target species (Franklin, 2010). Depending on the aims of an investigation and the type of data collected, several approaches are available to researchers. For many applications where surveys have been planned in advance of statistical analysis, standard methods such as generalized linear models, generalized additive models, and random forest are frequently used to model occurrence and abundance data (Elith et al., 2011).

However, given the time and cost of performing a systematic survey, especially in the marine environment, researchers often use archived datasets to perform modeling investigations (Araújo & Guisan, 2006). In many cases, these datasets only contain records on species presence, resulting in a situation where no absence data are available (Elith et al., 2011). For this reason, presence‐only models such as MaxEnt (Phillips, Anderson, & Schapire, 2006) have been developed that do not require absence data, but rather generate a large number of pseudo‐absences from the study area (Phillips et al., 2006).

While each approach outlined above has advantages, both fail to address the issue of detectability (Monk, 2014). Both approaches assume that detection probability is invariant, that is, that the target species is perfectly observed whenever it is present (Yackulic et al., 2013). This is often not the case when studying cryptic species and is an important consideration in the marine environment when sampling methods often do not result in direct observation of the study environment, for example, when using trawl or camera surveys (Monk, 2014). In a review of 108 articles that used MaxEnt, Yackulic et al. (2013) found that only 14% mentioned detection probability. This failure to address detectability introduces error to estimates of occurrence for the species being modeled and can result in erroneous reporting of covariate effects (Guillera‐Arroita, Lahoz‐Monfort, MacKenzie, Wintle, & McCarthy, 2014).

Site occupancy modeling (SOM) allows occupancy and detectability to be analyzed hierarchically as two separate processes, accounting for the problem of imperfect detection (MacKenzie et al., 2002). Monk (2014) provides a good overview of the need to adopt this class of model in the marine environment; while available for a similar period as MaxEnt, the SOM has received much less attention (e.g., Coggins, Bacheler, & Gwinn, 2014) in marine ecology investigations. The multiseason occupancy model (or dynamic occupancy model for its Bayesian counterpart (DOM)) allows occupancy, detection, local colonization, and extinction to be accounted for across several sampling seasons (MacKenzie, Nichols, Hines, Knutson, & Franklin, 2003). Seasons refer to primary sampling periods within which the population is assumed closed but between which the population can be subject to local extinction and colonization. The model assumes that at least one site has been visited more than once within a sampling period and that the true occupancy state is imperfectly observed, that is, it is a latent variable of the model. As such, the model can be thought of as a nonstandard GLMM with a binary random effect equal to 1 where the site is occupied by the target species and 0 where it is not (Kéry, 2010). Each of the four probabilities within the DOM (initial occupancy, colonization, extinction, and detection) can be modeled as a function of a set of covariate data or set as constant across sites within a given sample period (MacKenzie et al., 2003; Royle & Kéry, 2007). Where covariates are used to model probabilities, these must represent variability in the environment along temporal scales relevant to the phenomenon under study. Extinction and colonization effects, for example, require seasonally varying covariates. By contrast, detection effects require covariates that vary over much shorter temporal scales, allowing differences in detectability to be discerned within relatively short sampling periods. One possible reason why these models have not received as much attention in the marine environment as they have for terrestrial studies is the prohibitive cost of marine sampling. In order to satisfy the assumptions of the dynamic occupancy model, repeat visits are required within each of a number of seasons to collect both response and environmental data.

Covariates used for modeling that have been collected in situ at the time of making species observations are often assumed to better describe observed patterns in species distributions (Franklin, 2010). Recent research has shown, however, that this is not always the case and that modeling studies can benefit from a combination of in situ sampling and remote sensing data (Niedballa, Sollmann, Mohamed, Bender, & Wilting, 2015). Moreover, Newton‐Cross, White, and Harris (2007) demonstrated that remotely sensed or computer generated data can be more effective than data collected in situ for accurately predicting the occurrence of some terrestrial based species. Again, this is important in the marine environment where sampling is often expensive and time‐consuming compared to terrestrial studies. As such, marine researchers often have to rely on remote sensing (Brown, Sameoto, & Smith, 2012) and modeled data (Rattray, Ierodiaconou, & Womersley, 2015) to supplement in situ sampling. These latter datasets often come in the form of hydrodynamic models that mathematically represent tidal and wave forcing of the marine environment (Gunn & Stock‐Williams, 2013). While widely used in engineering and physical oceanography (e.g., Chen et al., 2011; McMillan & Lickley, 2008), their use in marine ecological investigations has been limited. This is likely due to the expense, in terms of time and computational power, of setting up a hydrodynamic model that accurately reflects conditions at spatial and temporal scales relevant to ecological processes and organism behavior.

This investigation aims, for the first time, to create a dynamic occupancy model to demonstrate the effectiveness of such an approach for a temperate marine fish species in a closed fishing area. The study generates unbiased estimates of occupancy and compares these to occupancy estimates obtained by survey methods alone. Inputs to the dynamic occupancy model comprise observed, derived, and simulated data. Observed data include observations of the target species, algae cover, and geomorphological complexity from video surveys; derived data include depth measured using multibeam echosounder (MBES) data, and terrain attributes derived from the MBES depth data. Additionally, the study makes novel use of current velocities simulated using a high‐resolution hydrodynamic model to demonstrate the utility of including these data in fisheries monitoring investigations.

## 2 METHODS

### 2.1 Analysis overview

**Figure 1:**
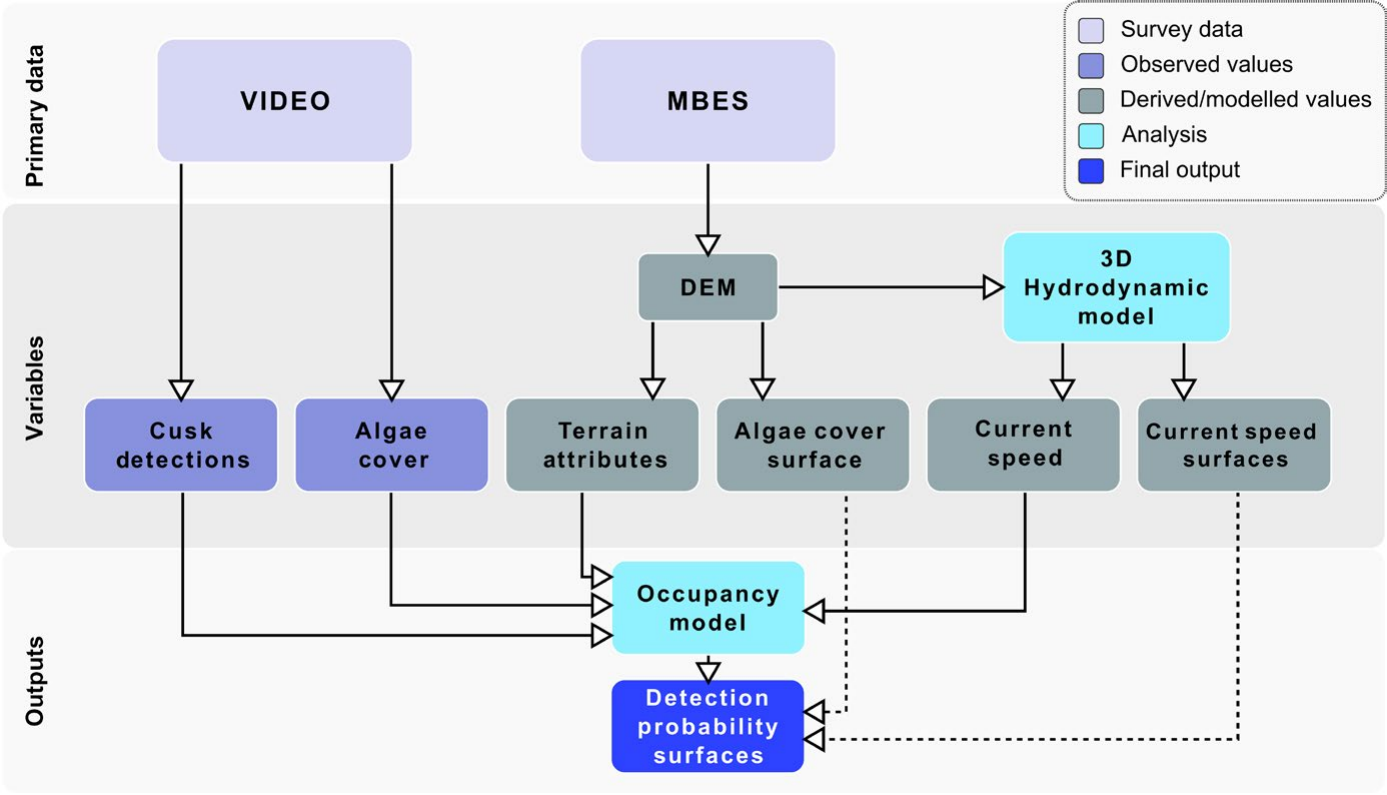
Flow diagram for analyses described in the methods. Solid lines represent the flow of information to create the dynamic occupancy model, and dashed lines represent the flow of information for producing the output probability surfaces in Figure 6

This study takes a multifaceted approach to producing a dynamic occupancy model (DOM) for a species of temperate marine fish at a remote rocky outcrop in the central Gulf of Maine. The model was specified in a Bayesian framework to account for a small amount of separation in the detection data and to allow the use of a finite sample estimator that generates more accurate estimates of occupancy for a small sample size (Royle & Kéry, 2007). The analysis used data observed or derived from two primary datasets: a series of noninvasive underwater video surveys collected over four sampling seasons and a MBES survey conducted for the Gulf of Maine mapping initiative (SAIC, 2005). Observations of the response, cusk, were made using the video footage. During each video survey, detections of cusk were recorded along with the time of the observation. Simultaneous observations of algae cover and morphological complexity were made from the video footage, to be later used as explanatory variables. Separately, the digital elevation model extracted from the MBES data was used for three purposes: (a) to derive terrain attributes to assess morphological complexity over the study site; (b) to create a surface of algae cover over the study site based on empirical extinction depths; and (c) as an input to a standalone hydrodynamic model. The hydrodynamic model was used to estimate bottom current conditions at the study site, from which two outputs were generated (a) point estimates of time‐varying bottom current speed at each of the video locations surveyed for cusk and (b) time‐varying surfaces of current speed for the entire study area. Once all primary data had been processed, the DOM was created. Model coefficients for the significant terms in the detection submodel of the DOM (observed algae cover and bottom current point estimates) were obtained and used to generate the final outputs. This was achieved by predicting the model coefficients over the algae cover surface and bottom current surfaces created above, creating spatio‐temporally varying predictions of detection probability for the study area. Each step of the analysis is described in full in the following sections (Figure 1).

### 2.2 Candidate species and area

The candidate species for this investigation is cusk (*Brosme brosme*, Lotidae); a cryptic, bottom dwelling species found in the eastern and western Atlantic Ocean and designated a species of concern by the National Oceanic and Atmospheric Administration (NOAA) National Marine Fisheries Service (NOAA 2009). In the western Atlantic, cusk are found from Nova Scotia in Canada to New Jersey in the USA and typically stay in deeper waters (>100 m) in these areas. Within the Gulf of Maine, however, they are typically found in shallower water owing to the relatively shallow depths of the internal Gulf (Bigelow & Schroeder, 1953). While relatively little is known about their specific life history and ecology (Davies & Jonsen, 2011), it has been noted that cusk prefer structured habitat and use kelp forests, boulder piles, and rock crevices as refugia (Auster & Lindholm, 2005; Hare et al., 2012). In addition, they are considered to be weak swimmers (Bigelow & Schroeder, 1953), so make an ideal species for study on how their behavior is affected by variability in movements of the water column. The species also has a small home range (Dultz, 2013), making it suited to the assumptions of the dynamic occupancy model.

**Figure 2:**
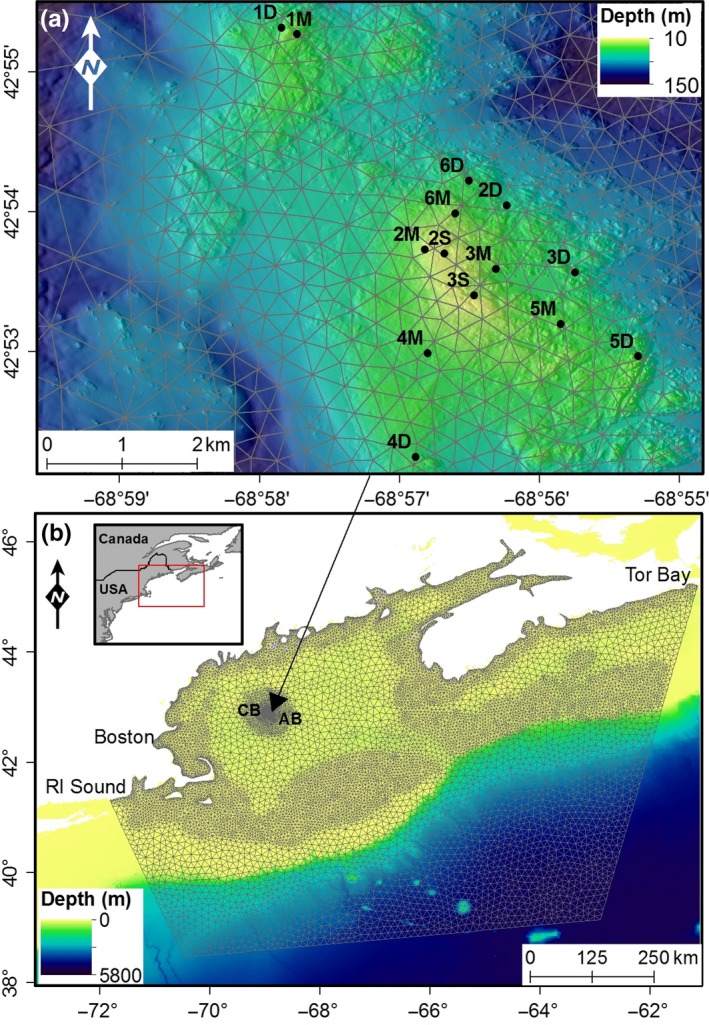
(a) Location of 14 sites sampled for cusk at Cashes Ledge. Gray triangles are from the hydrodynamic model computational mesh, the full domain of which is in (b) along the location of Cashes Ledge in the Gulf of Maine. CB is Cashes Basin, AB is Ammen Basin

Data for the investigation were collected at Cashes Ledge in the central Gulf of Maine, approximately 170 km northeast of Boston (Figure 2a). The Ledge has been closed to bottom tending fishing gears since 2002 (Sherwood & Grabowski, 2016) and supports large resident populations of several commercially important fish species (Grabowski, McGonigle, & Brown, 2010). The site displays a tripartite zonation of macroalgae around the summit with each of the three zones reaching record depths for boreal‐subarctic waters: leathery macrophytes to 40 m, foliose red algae to 50 m, and crustose algae to 63 m (Vadas & Steneck, 1988). The Ledge comprises morphologically complex granite shoaling at 10 m water depth, with sand and gravel deposits appearing around 60 m and silt dominated habitats below 80 m. To the east and west, the site is flanked by relatively deep basins (<220 m) dominated by sands, fine silt, and clay (Uchupi & Bolmer, 2008).

### 2.3 Data collection and covariate generation

Underwater video surveys were conducted at 14 sites on Cashes Ledge by the Gulf of Maine Research Institute using a drop camera in summer 2006 and spring, summer, and autumn 2007. Sites are defined here as compact geographical areas wherein samples are less than 30 m apart and features of the abiotic environment are homogenous. Sampling was stratified by depth to include two shallow sites (<20 m), six intermediate sites (20–40 m), and six deep sites (>40 m). During each sampling season, a maximum of three replicate surveys were conducted at each site, with an average of two. Camera units were deployed and left in situ for up to 1.5 hr during each survey, recording the time in and position of the camera. The camera was mounted on the sampling equipment such that the field of view was parallel to the seabed; no directional controls were in place, so the azimuthal direction varied between samples. For full camera set up see Grabowski et al. (2010). Any samples where the camera equipment landed with the camera facing into the water column and therefore unable to view the seabed were discarded and not used in any further analyses. After checking each sample for positional accuracy, videos were examined noting the time on the video any cusk were observed. For videos where cusk were present, the video time was combined with the survey start time to obtain the exact time of the observation. Where cusk were absent in a video, a time was randomly sampled from the length of the video to obtain a time for the null observation.

In addition to the observations of cusk, the videos were used to qualitatively assess fine‐scale morphological complexity (high, moderate, low) and algae density. Algae density was used to classify the study area into areas of two different bottom types according to algae cover, henceforth bottom type: areas with canopy forming species and areas with no canopy forming species. Sampling effort was defined simply as the length of bottom time in each video. Depth for each observation was obtained from 5 m resolution MBES data collected for the Gulf of Maine Mapping Initiative (SAIC 2005). Morphological complexity was derived from the MBES data using the relative deviation from the mean value (RDMV) as recommended by Lecours, Devillers, Simms, Lucieer, and Brown (2017) in a 3 × 3 cell moving window. A breakdown of the mean, minimum, and maximum values for each of the depth strata sampled is in Table 1.

**Table 1:** Mean, maximum, and minimum for each of the covariates falling within the depth strata sampled

Covariate	Stratum	Min	Mean	Max	Number
Depth	Shallow	−23	−17	−11	—
	Medium	−43	−33	−25	—
	Deep	−57	−49	−39	—
Current speed	Shallow	0.05	0.11	0.17	—
	Medium	0.03	0.09	0.19	—
	Deep	0.01	0.09	0.2	—
CurMax	Shallow	0.21	0.22	0.24	—
	Medium	0.16	0.19	0.23	—
	Deep	0.16	0.19	0.22	—
RDMV	Shallow	0.14	0.56	1.39	—
	Medium	0.06	0.31	0.36	—
	Deep	0.08	0.25	0.67	—
Algae cover: none	Shallow	—	—	—	0
	Medium	—	—	—	10
	Deep	—	—	—	45
Algae cover: canopy	Shallow	—	—	—	21
	Medium	—	—	—	36
	Deep	—	—	—	0

Note

Values for algae cover are reported as counts, as these are categorical variables. “—” indicates no relevant data available.

### 2.4 Hydrodynamic model

Hydrodynamics were assessed using a coupled wave‐current model produced using MIKE by DHI (Danish Hydraulic Institute, 2014). The current model solves the three‐dimensional incompressible Reynolds averaged Navier‐Stokes equations, while the wave model solves a fully spectral wind‐swell formulation. The models are coupled to include wave‐current interactions and are solved using a finite volume method over a flexible mesh that allows higher resolution in areas of interest (Danish Hydraulic Institute, 2014).

The domain for the model (Figure 2b) incorporated the Gulf of Maine from Tor Bay to Rhode Island and extends seaward off the continental shelf to allow the Gulf to respond freely to tidal forcing (McMillan & Lickley, 2008). Forcing was supplied to the model as spatially and temporally varying surface elevation from the DTU10 0.125° global tidal model (Cheng & Andersen, 2010). Calibration of the model was achieved by adjusting the value of bed resistance over a series of 13‐month simulations (one‐month warm‐up, 12‐month usable data). After each calibration simulation, harmonic analysis was conducted for 67 sites within the Gulf of Maine for comparison against empirical data from Moody et al. (1984). Where disagreement between known and modeled data was unacceptably large, values of bed resistance were iteratively adjusted to fine‐tune the harmonics.

Once the model harmonics were calibrated to maximally correspond to empirical data, the computational mesh was refined to increase resolution at Cashes Ledge. Here, the maximum horizontal resolution was 135 m with an average horizontal resolution of 220 m. In addition to refining the model mesh, atmospheric forcing was introduced to the model using the National Centres for Environmental Prediction (NCEP) Climate Forecast System Reanalysis (CSFR) 6‐hourly, 0.5° global weather model (Saha et al., 2010, 2011). Model validation was subsequently conducted on the refined mesh model by hindcasting periods of time not included in the calibration models. Validation included the assessment of model outputs against measured wind, wave, and current data. The model was considered validated when outputs were minimally different to measured data. All model runs were performed with a time step of 20 min to allow validation against measured data.

Full details of the model setup, calibration, and validation can be found in the Supporting Information Appendix S1. Once the model had been validated, current speeds were extracted from the water‐seabed interface layer to capture current variations on the bottom. Times for the observations of cusk were then matched to the temporally closest value of current speed. Visualization of temporal variability in current magnitude and direction was achieved using a tidal ellipse created using MATLAB (Xu, 2002). The ellipse was derived for a point in open water 100 m from the summit in order to capture the movement of water without influence from local topography.

### 2.5 Dynamic occupancy model

The model was specified using notation from Royle and Kéry (2007) where ψ_*t*_ is the probability of initial occupancy at time period *t*,* ϕ*_*t*_ is the probability that a site remains occupied between *t* and *t *+* *1, *γ*_*t*_ is the probability that a site is colonized between *t* and *t *+* *1, and *p* is the probability of detection. During data exploration boxplots revealed a high degree of collinearity between RDMV and fine‐scale complexity observed in the video data. Fine‐scale complexity was excluded from further analyses; it is a categorical variable so including it would require estimation of more model parameters. Covariates tested for *ψ*_*t*_ were depth, fine‐scale morphological complexity, RDMV, maximum current speed throughout the sampling season (CurMax) and CurMax^2^. Depth, fine‐scale complexity, and RDMV as several studies have shown the importance of depth and seabed roughness for cusk habitat selection (Davies & Jonsen, 2011; Hare et al., 2012; Knutsen et al., 2009); CurMax and CurMax^2^ to test for any limiting effect of hydrodynamic forcing on cusk habitat selection. Covariate effects were not included for *ϕ*_*t*_ or *γ*_*t*_ and were therefore assumed to be the same for all sites within each sampling period (MacKenzie et al., 2003; Royle & Kéry, 2007). Covariates included for *p* in the detection submodel were current speed, sampling effort, and bottom type. Current speed as cusk are weak swimmers whose movement is likely to be affected by movements of the water column; sampling effort as the longer the camera is in the water, the more likely it is that a fish will be observed in any given sample; bottom type as canopy forming species are likely to obscure vision and therefore affect detectability of cusk.

Covariate effects for each submodel were transformed using a logit link function, and all continuous covariates were standardized before analysis. In order to simplify model specification two models were assessed, one without (A) and one with (B) covariates for ψ, both of which included covariates for *p*.

Specification for *ψ* in model B:

$$\text{logit}\left(\psi \right)=\beta_{1}+\beta_{2}\cdot \text{Depth}+\beta_{3}\cdot \text{RDMV}+\beta_{4}\cdot \text{Current Max}+\beta_{5}\cdot {\text{Current Max}}^{2}$$

where *β*_*i*_ are the regression coefficients, RDMV is the relative deviation from the mean value, and current max is the maximum current speed at the site in any of the sampling seasons.

Specification for *p* in models A and B:

$$\text{logit}\left(p\right)=\alpha_{1}+\alpha_{2}\cdot \text{Current}+\alpha_{3}\cdot \text{none}+\alpha_{4}\cdot \text{Current}|\text{none}$$

where current is the current speed at the time of sampling for cusk, none is the bottom type with no canopy forming algae, and Current|none is the interaction between current speed and the bottom type with no canopy forming algae.

### 2.6 Model validation and outputs

Goodness of fit (GOF) for each model was assessed using Bayesian *p*‐values; for each draw of the MCMC algorithm, new data were simulated given the set of parameters estimated by the model, and Pearson residuals were calculated for each observed and simulated data point. The Bayesian *p*‐value is the proportion of times the simulated residual is greater than the observed residual, with values closer to 0.5 indicating better fit (Kéry, 2010).

For the *k* covariates in each model, importance was tested using binary inclusion variables *w*_*k*_ ~ Bernoulli(0.5). Averaging over the posterior distribution of *w*_*k*_ gives the probability that term *k* belongs in the model, with values closer to 1 indicating a higher inclusion probability. The inclusion variable for interaction terms was defined as the product of itself, and the component inclusion variables as recommended by Kruschke (2014) ensuring the interaction was only assessed when the lower order terms were included. Covariates and interactions were kept in the model when their inclusion variable had a posterior mean greater than 0.5 (Coggins et al., 2014).

All logit scale coefficients were given weakly informative t‐distribution priors (*μ* = 0, *σ* = 1.566, *v *=* *7.763) as recommended by (Dorazio, Gotelli, & Ellison, 2011), while all nonvarying probabilities (*ϕ*,* γ*) were given uniform priors from 0 to 1. Sensitivity of the posterior distribution of parameter estimates was assessed following Dorazio et al. (2011) using three prior distributions recommended for logistic regression; Jeffreys prior (Firth, 1993), *t*‐distribution with *μ* = 0, *σ* = 2.5, *v *=* *7; those of Gelman, Jakulin, Pittau, and Su (2008), *t*‐distribution with *μ* = 0, *σ* = 2.5, *v *=* *1; and those of Dorazio et al. (2011), as detailed above. Identifiability of parameter estimates was assessed by plotting and calculating the amount of overlap between the prior and posterior distributions. These were considered identifiable if the overlap was below 35% (Garrett & Zeger, 2000; Gimenez, Morgan, & Brooks, 2009). The MCMC algorithm was set up with 5 chains, each sampling 50,000 draws. The first 10,000 draws were discarded as a burn‐in period, and every tenth sample was stored for analysis. Chains were assessed visually for mixing and autocorrelation, and convergence was assessed using the Gelman‐Ruben diagnostic (Gelman & Rubin, 1992) with values less than 1.1 considered converged. Occupancy was determined using a finite sample estimator that is recommended for a small number of nonrandomly selected sites (Royle & Kéry, 2007). This estimator allows a distinction to be made between estimates derived for parameters of the population and those derived for sites in the actual sample. The finite sample estimator reduces the variance of the point estimates generated for the sampled sites and is an additional benefit of fitting the model in a Bayesian framework (Royle & Kéry, 2007).

Continuous surfaces of modeled current speed were generated from the validated hydrodynamic model to visualize spatio‐temporal variability of current speed along a tidal cycle at Cashes Ledge. Predictions of bottom type were made based on the extinction depths of various algae previously reported at the Ledge (Vadas & Steneck, 1988). Surfaces of detection probability were then generated for the entire Ledge for the tidal cycle by predicting the occupancy model coefficients over the current speed and bottom type surfaces.

All statistical analyses and predictive mapping were carried out in the R environment (R Core Team 2015), and Bayesian inference was conducted in JAGS (Plummer, 2009).

## 3 RESULTS

### 3.1 Cusk observations and data exploration

Of 112 replicate surveys analyzed, 39 contained cusk and 73 did not. These correspond to 21 shallow samples, 46 intermediate samples, and 45 deep samples representing average depths of 17 m, 33 m, and 49 m, respectively. Analysis showed 57 video surveys were conducted in the canopy forming region and 55 in the noncanopy forming region.

**Figure 3:**
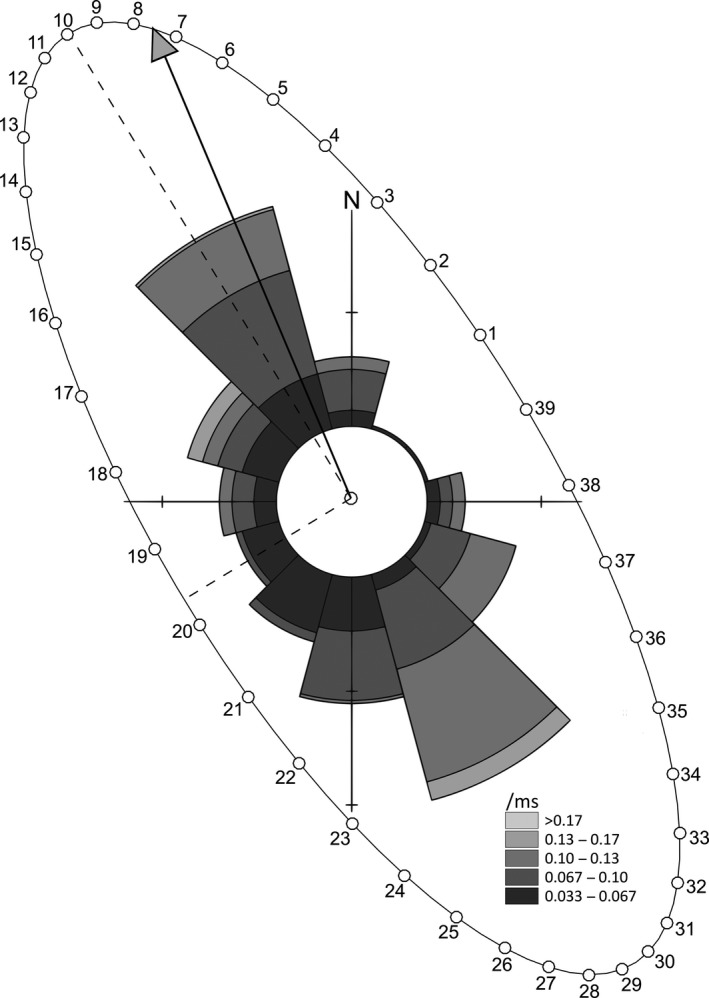
Tidal ellipse for a point in open water 100 m away from Cashes Ledge. The distance from the centroid to the arc of the ellipse is proportional to the current speed, and the direction of a vector radiating from the centroid to any point on the arc represents the direction any that point. The numbers on the arc correspond to the numbers on the tidal curve and in Figure 6

**Figure 4:**
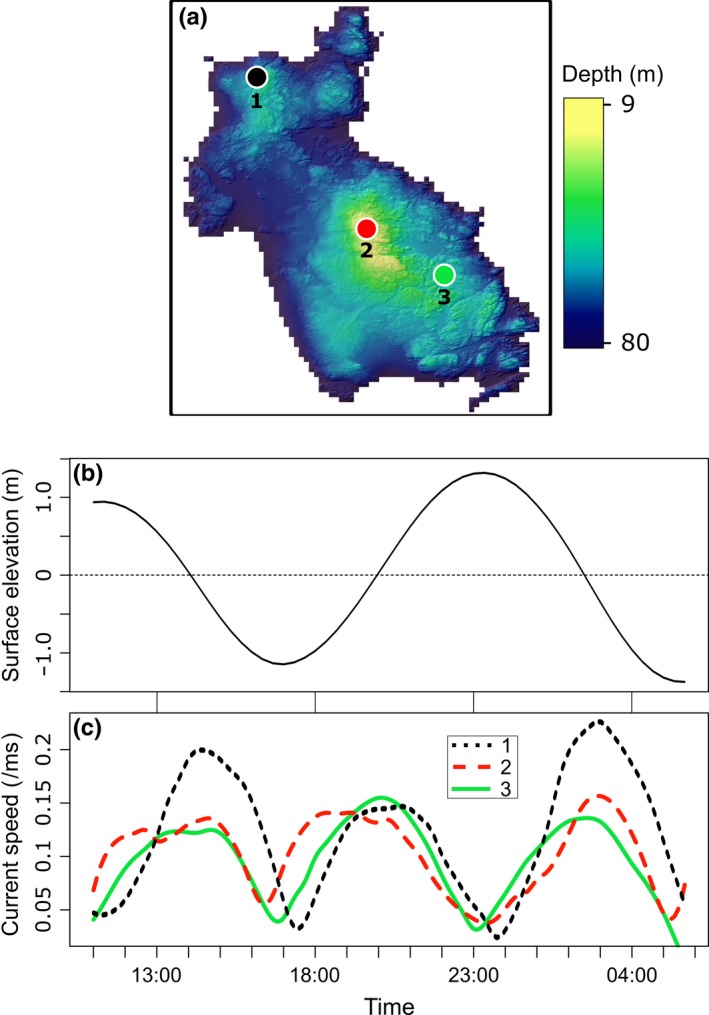
Example of difference in arrival time of tidal signal for three sites, one deep (1), one intermediate (3) and one shallow (2) at Cashes Ledge over one and a half tidal cycles throughout the summer 2006 sampling season. Time on the *x*‐axis of the tidal curve (b) and current speed plot (c) are the same. The locations of the three sites are marked on the map (a)

Harmonic analysis of the hydrodynamic model reveals accuracy of 3.8 cm mean absolute deviation in amplitude and 4.6° in Greenwich phase lag for the M_2_ tidal constituent. Bottom current speeds at Cashes Ledge range from 0.01 ms^−1^ to 0.31 ms^−1^ throughout the four sampling periods, with a maximum of 0.21 ms^−1^ while the camera equipment was deployed. The tidal ellipse and current rose show that maximum flow occurs in a north–south orientation along the semimajor axis of the ellipse (Figure 3). A short time lag of around 1 hr is observed in the arrival time of the tidal signal between the north and south of the Ledge (Figure 4).

### 3.2 Model validation and outputs

Bayesian p‐values were 0.58 for model A (Table 2, top) and 0.62 for model B (Table 2, bottom). Sensitivity analysis showed no significant effect of choice of prior on posterior distributions for all parameters excluding those for the covariates for *ψ* in model B. The overlap of the posterior distributions of parameters in model A with their prior distributions ranged from 20% to 31%, with the highest amount of overlap for the estimate for sampling effort. All parameters for this model were therefore considered identifiable. Inclusion variables for model A indicated that current speed, bottom type, and the interaction between current speed and bottom type should be included in the detection submodel (Table 2). Inclusion variables for model B indicate less than or equal to 50% probability that any covariates belonged in the occupancy submodel and again that current speed, bottom type, and the interaction term between current speed and bottom type should be included in the detection submodel (Table 2).

**Table 2:** Parameters estimated for coefficients for occupancy model A (without covariates for ψ) and model B (with covariates for ψ)

Model A	GOF	Gelman‐Ruben MV					
	0.58	1.01			Credible Interval		
Sub‐model	Parameter		Mean	s.d.	2.50%	97.50%	Inclusion probability
Detection	Intercept		−1.57	0.79	−3.27	−0.04	–
	Current		−1.26	0.59	−2.58	−0.19	0.66
	Effort		−0.37	0.75	−1.94	1.05	0.32
	none		3.01	0.88	1.52	5.19	0.99
	Current|none		1.77	0.71	0.44	3.32	0.79
**Model B**	**GOF**	**Gelman**−**Ruben MV**					
	0.62	1.01			**Credible interval**		
**Sub‐model**	**Parameter**		**Mean**	**s.d**	**2.50%**	**97.50%**	**Inclusion probability**
Detection	Intercept		−1.43	0.83	−3.17	0.2	–
	Current		−1.31	0.63	−2.67	−0.21	0.7
	Effort		−0.35	0.79	−1.97	1.09	0.34
	none		2.85	0.93	1.3	4.98	0.99
	Current|none		1.85	0.75	0.47	3.44	0.82
Occupancy	Intercept		1.41	1.4	−0.93	4.55	–
	Depth		−1.66	1.55	−5.08	1.24	0.5
	Complexity		0.99	1.66	−2.01	4.63	0.49
	Current max		0.04	1.31	−2.53	2.64	0.4
	Current max^2^		0.17	1.31	−2.42	2.78	0.39

Note

GOF is the Bayesian *p*‐value, where values closer to 0.5 indicate better fit. Gelman‐Ruben MV is the multivariate Gelman‐Ruben convergence statistic, where values close to 1 mean the model has successfully converged. Current is the current speed at the time of observation of cusk, effort is sampling effort, none is regions with no canopy forming species of algae, current|none is the interaction between current and none, complexity is RDMV, current max is the maximum current speed during the sampling season.

**Figure 5:**
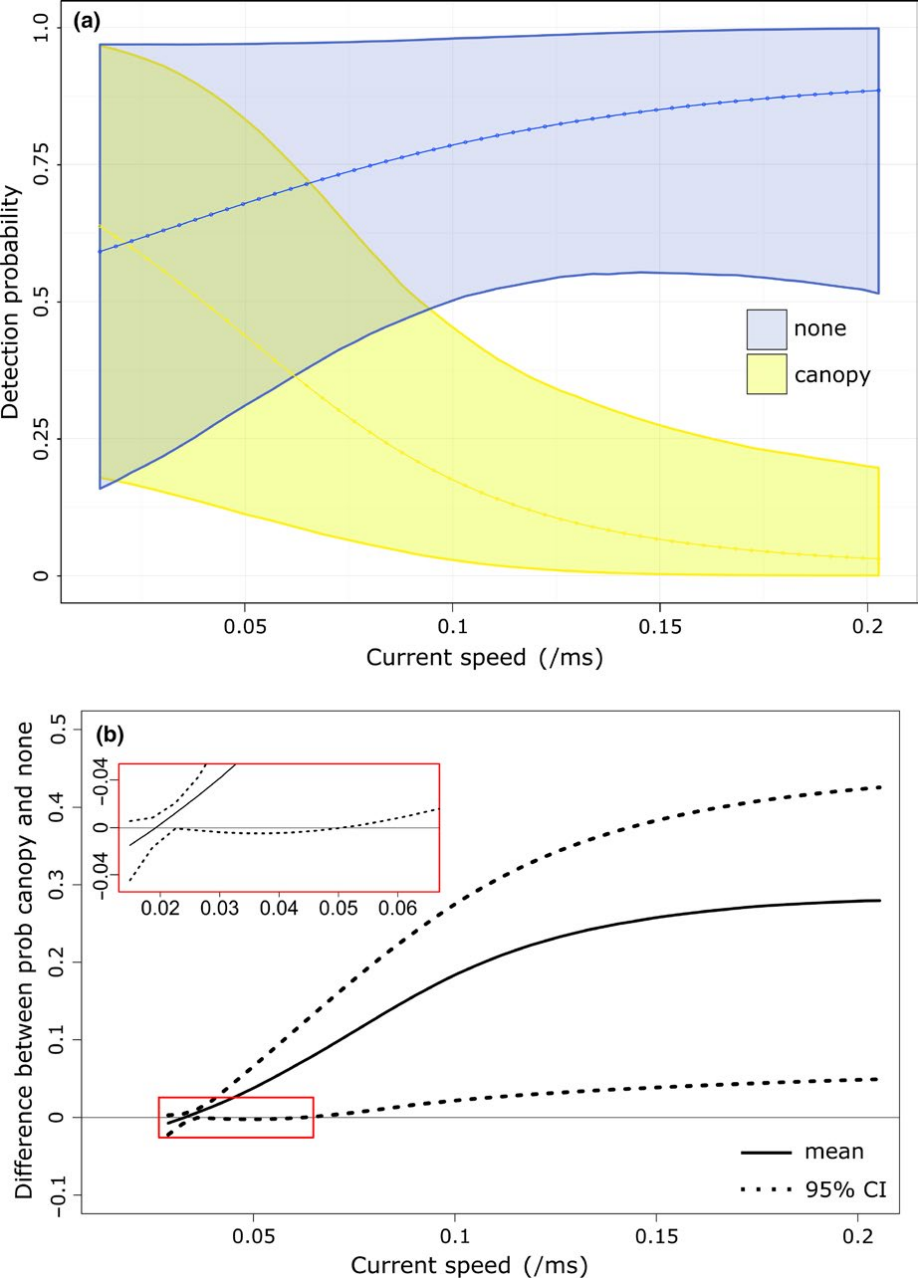
Model outputs showing (a) final model for detectability of cusk at Cashes Ledge based on current speed and bottom type, colored bands are 95% credible interval, (b) difference in detection probability between canopy forming and noncanopy forming regions. Where the 95% credible interval includes zero, there is no significant difference in detection probability

Results for model A show that with increasing current speed, detection rates in the two bottom types diverge: increasing in the noncanopy forming region and decreasing in the canopy forming region (Figure 5a). At low current speeds (≈1 cm/s), the probability of detecting cusk is almost identical in both bottom types, and there is no credible difference in detection probability between 0 cm/s and around 5 cm/s among bottom types (Figure 5b and inset).

**Figure 6:**
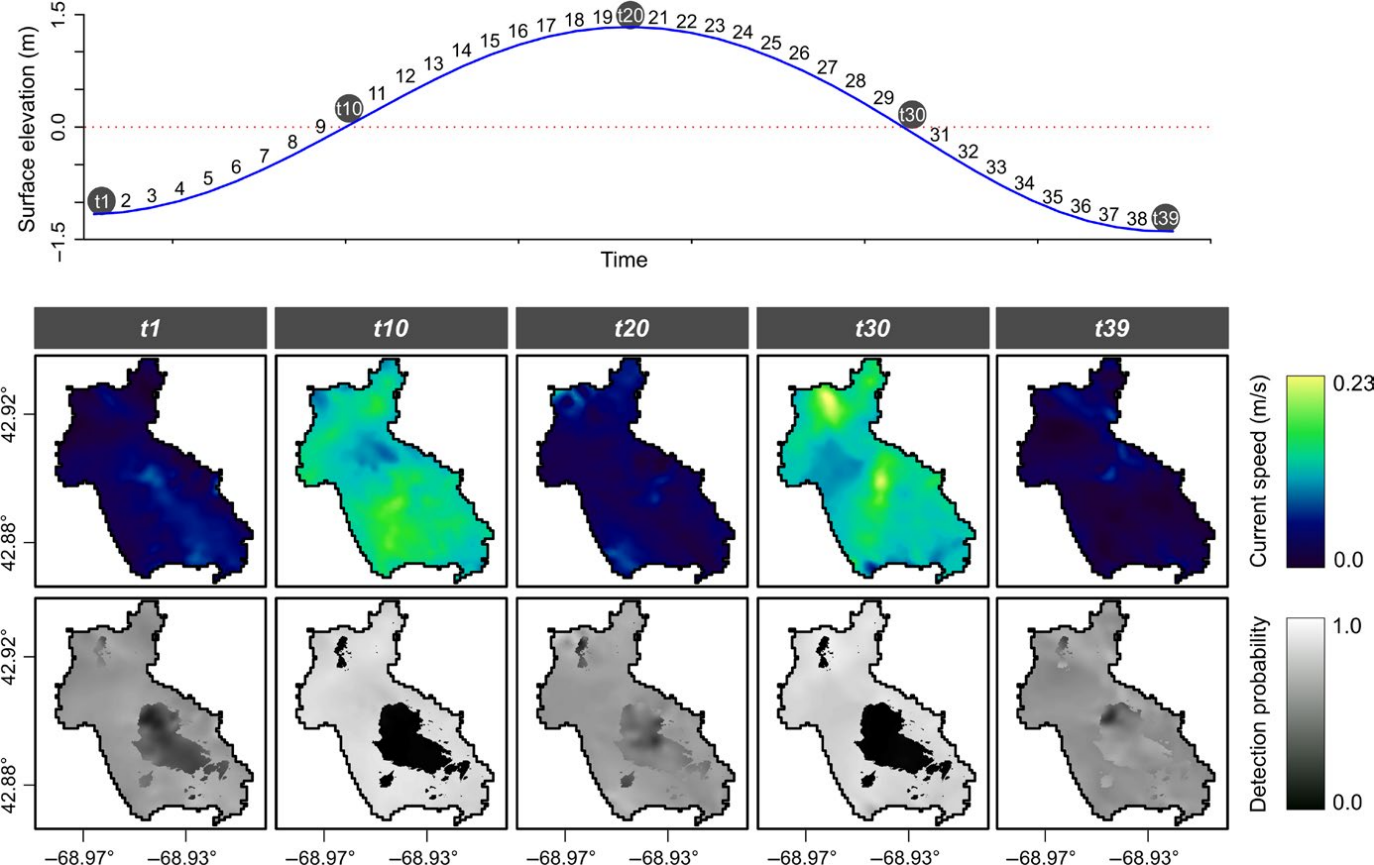
Tidal curve (top), current speeds (middle) and predicted probability surfaces (bottom) along a full tidal cycle during the summer 2006 sampling season. Surfaces are predicted from the detection model outputs. Numbers of each panel in the gray boxes (*t*1–*t*39) correspond to the numbers on the tidal curve. The darker regions in *t*10 and *t*30 indicate the areas with canopy forming species of algae based on extinction depth for those species at Cashes Ledge, while the lighter regions are the areas with no canopy forming species of algae

Predictions of detection probability reveal similar rates of detectability in both bottom types at low and high tide (Figures 6.t1 and 6.t39), while varying significantly between these times (Figures 6.t10, 6.t20 and 6.t30).

**Figure 7:**
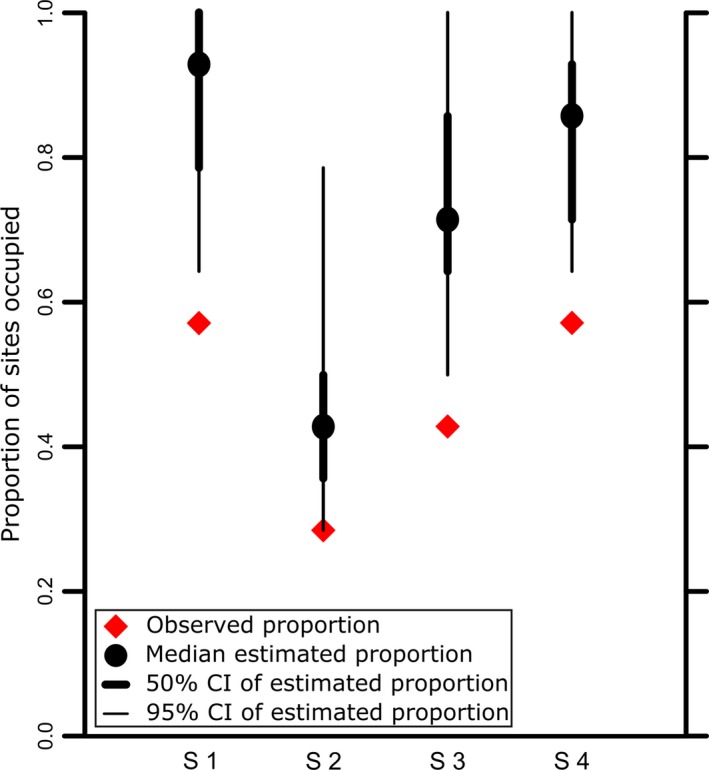
Observed and median estimated proportions of sites occupied by cusk at Cashes Ledge during the four sampling periods; S1 (Summer 2006), S2 (Spring 2007), S3 (Summer 2007 and S4 (Autumn 2007). Also shown are the 50% and 95% credible intervals for the posterior estimates of the proportion of occupied sites

Estimates of the proportion of sites occupied by the finite sample estimator are 0.88 (95% CI: 0.64–1.0), 0.45 (95% CI: 0.29–0.79), 0.74 (95% CI: 0.50–1.0), and 0.83 (95% CI: 0.64–1.0) for seasons 1, 2, 3, and 4, respectively. This compares to observed proportions of 0.57, 0.28, 0.43, and 0.57 for seasons 1, 2, 3, and 4, respectively (Figure 7). Colonization probabilities for all sites are 0.52, 0.77, and 0.55 for colonization between seasons 1–2, 2–3, and 3–4, respectively. Extinction probabilities are 0.56, 0.44, and 0.15 for extinction between seasons 1–2, 2–3, and 3–4, respectively; these probabilities are the compliment of *ϕ* in the parametrization described in the methods. Population growth is estimated at 0.56 between season 1 and 2, 1.74 between season 2 and 3, and 1.15 between season 3 and 4. This represents a decrease in the number of occupied sites from summer 2006 to spring 2007, an increase from spring to summer 2007, and an increase from summer to autumn 2007. JAGS code for the final model is given in the Supporting Information Appendix S2.

## 4 DISCUSSION

The problem of bias introduced by failing to account for detectability of mobile fish species when estimating their occupancy was addressed in this study. Using a dynamic occupancy model and treating the true occupancy state as a latent variable of the model, occupancy estimates are significantly greater than occupancy assessed from observation alone. Taking account of imperfect detection, the difference in estimated and observed proportion of sites occupied by cusk range from 0.17 to 0.31. This highlights the need to incorporate detectability of the target species into species distribution modeling efforts (Rota, Fletcher, Evans, & Hutto, 2011).

### 4.1 Cusk behavior

Cusk are slow moving weak swimmers (Bigelow & Schroeder, 1953; COSEWIC 2003), and it is therefore not unreasonable to expect them to be influenced by hydrodynamic conditions. Outputs of model A show that, indeed, cusk detectability is affected by changes in current speed along a tidal cycle. Within the noncanopy forming regions of Cashes Ledge, the increase in detectability with increasing current speed can be thought of as a proxy for increased activity of cusk. This increase in activity is interpreted in one of two ways, either as cusk searching for morphologically complex environments as refuge, or as cusk using the movement of water as an opportunity to forage for food. Cusk have been observed to prey on cunner (*Tautogolabrus adspersus*; Auster & Lindholm, 2005), and cunner in turn have been observed to forage more on exposed surfaces near refugia with increasing current velocity (Auster, 1988, 1989).

Other than these prey, little is known about cusk diet in the western Atlantic. In European waters, however, stomach contents analysis shows that cusk will eat a range of crustaceans and molluscs, both of which have been found in abundance in the shallower kelp dominated regions at Cashes Ledge (Vadas & Steneck, 1988; Witman & Sebens, 1992). Within this canopy forming algae region, as current speed begins to increase, detection probability decreases quite rapidly. While marine fish have been shown to use kelp habitats as both refuge and foraging grounds (Holbrook, Carr, Schmitt, & Coyer, 1990; Uhl, Bartsch, & Oppelt, 2016), the presence of canopy forming species of algae will encumber detectability, especially in relatively high flows or with high energy wave conditions (Rattray et al., 2015).

In previous studies of cusk habitat usage, the species has been recorded at much greater depths than those observed in this study (Davies & Jonsen, 2011; Hare et al., 2012; Nye, Link, Hare, & Overholtz, 2009). One explanation of the depths observed in this study is that the fish in this region are year round residents that have become accustomed to living at comparatively shallower depths. Bigelow and Schroeder (1953) note that cusk do not often move from bank to bank, and no seasonal spawning migrations have been noted for the species (COSEWIC 2003). The spawning season for cusk in the Gulf of Maine extends from April to July (Bigelow & Schroeder, 1953), and this might explain the large growth rate in the number of sites occupied between Spring 2007 and Summer 2007 (sampling seasons 2 and 3 respectively) as fish become more active in search of a mating partner. These two time periods together take in portions of the spawning season for cusk; it is therefore possible that the later it is in the season, the more active the fish are in their search.

An assumption of the dynamic occupancy model is that within a primary sampling season the population remains closed, but can be open to local extinction and colonization between seasons (Kéry, 2010). Cusk are described as a “station keeping bottom” or “station keeping cover” species (Auster & Lindholm, 2005), and evidence suggests that the home range of cusk is small (Dultz, 2013). Furthermore, cusk show strong affinity for complex habitats while avoiding substrata with no structure (Bigelow & Schroeder, 1953; COSEWIC 2003). Cashes Ledge is surrounded on all sides by a number of deep basins, most notably Ammen Basin to the east and Cashes Basin to the west (Figure 2), which consist of unconsolidated substrata (Uchupi, 1966; Uchupi & Bolmer, 2008). As such, the assumptions of the model are satisfied; cusk remain on station and are kept on Cashes Ledge by expanses of nonfavorable habitat on all sides.

Throughout the four sampling seasons within this study, the same camera setup was used. No variability in detection probability should therefore be expected due to gear differences. In investigations using camera surveys combined with fish traps to assess detectability using simultaneous data collection methods (Coggins et al., 2014), the approach of adding cameras to other gear types has been recommended (Bacheler et al., 2014). In the current study, the camera system was used in isolation, and given the limited field of view and a lack of control over orientation once on the seabed, detection probabilities are expected to be and are less than one.

No significant effects were observed for any of the covariates tested in the initial occupancy submodel of model B. While it is likely that some covariates are missing from this submodel, due to the small sample size, it is not possible to add more terms without overfitting the model. The covariates that were included were of importance in two ways. First, they were important in terms of what has previously been reported as driving cusk habitat: depth and surface complexity (Hare et al., 2012). Secondly, maximum current speed and maximum current speed squared were included to test whether hydrodynamics play a role not only in determining cusk behavior, but also in limiting cusk habitat choice. Nevertheless, the main purpose of this study was to give consideration to the detectability issue in marine fish occupancy modeling. Given that the dynamic occupancy model is able to handle constant initial occupancy probabilities across all sites in the study domain, this lack of fit for the initial occupancy state does not present problems for inference about detectability.

### 4.2 Recommendations for future studies

In this study, detection probability ranged from 0.59 to 0.88 in the noncanopy forming region and from 0.03 to 0.64 in the canopy forming algae region. These results are broadly comparable to detection probabilities from other studies using camera surveys with other modeling approaches to detect marine fish (Bacheler et al., 2014; Coggins et al., 2014). These detection probabilities are conditional on cusk being present at the site being observed and also need to be considered in light of the fact that bottom time for the camera was relatively high in this investigation. It should also be noted that, for some species of fish, the presence of camera equipment on the seabed may encourage more curious individuals to investigate so may introduce some bias to the results (Stoner, Ryer, Parker, Auster, & Wakefield, 2008). In both models assessed in this study, the relationship between sampling effort and cusk detection was found to be insignificant. The term was left in the models, however, due to its theoretical importance; one of the most important factors affecting the detection of any organism is the amount of effort put into trying to detect it. Especially when dealing with small sample sizes, insignificant covariates should be left in models when they are theoretically important (Schuenemeyer & Drew, 2010).

While most of the spatial variability in detection probability can be explained by tidal phase, some small differences persist within each of the bottom types in Figure 6. These can be explained by local differences in current speed caused by water movement around topographic features on the seabed. Differences in detection probability created by these local variations in current speed in the deeper regions, and to a lesser extent in the shallow regions, are better understood when flow direction and topography are considered. Given that the strongest flows indicated by the tidal ellipse occur in a northerly and southerly direction (Figure 3) and that the orientation of the Ledge is approximately SE–NW (Figure 4c), it follows that different areas of the Ledge will experience maximal flow at different stages of the tidal cycle. Similarly, minimum flow occurs at different times throughout the 12.4 hr M_2_ tidal period (Figure 3). This has an effect on the arrival time of the increase in current speed at different locations throughout the study area (Figure 4b). Failing to take these local differences into consideration can have consequences when trying to plan similar surveys when the target species may be affected by flow rates. Such camera surveys are often the most suitable method for monitoring marine reserves and areas closed to mobile fishing gears as they are considered both cost effective and noninvasive (Bouchet & Meeuwig, 2015; DeCelles, Keiley, Lowery, Calabrese, & Stokesbury, 2017). The need to consider detectability is therefore paramount to obtaining unbiased results.

It is recognized that organisms living in marine environments exist in a multidimensional space, where even though they may live on the seabed, the constant flux of the water column plays an important role in shaping their distributions (Brown, Smith, Lawton, & Anderson, 2011). While the same is true in the terrestrial environment (Jung, Kaiser, Böhm, Nieschulze, & Kalko, 2012), the data needed to describe the nature of the ocean are often harder to collect or generate. Broad scale covariates used in marine investigations, such as temperature, current speed and direction, often come from depth averaged, or surface values of the covariate of concern. Additionally, if these covariates are not collected at the time of survey as is often the case, they need to be found as records elsewhere, or modeled using an appropriate method. Temperature, for example, is known to affect the metabolism and behavior of marine fish (Biro, Beckmann, & Stamps, 2010; Remen et al., 2015), which can affect the detection probability. Including temperature in the hydrodynamic model used in the current study can only be achieved by specifying water densities as a function of temperature and salinity. This presents significant challenges in coastal environments where highly variable surface conditions due to fresh water inputs cause errors in modeled hydrodynamics. As a result, temperature data were available only as low resolution depth averaged values, providing information important only to seasonal variations in temperature. Any variability in cusk occupancy due to seasonal temperature variations would already have been captured in the occupancy model by the latent variables for gamma and phi. Nevertheless, the inclusion of temperature data in future studies could potentially provide more insight into variability in fish behavior and detection as a result of thermal stresses. Hydrodynamic information in this investigation came from a 3‐dimensional hindcast model of wave and current conditions at Cashes Ledge and explained ecologically relevant phenomena that may otherwise have been overlooked. While forecasting these types of data may not be a viable option for many researchers planning future studies, it is a recommendation of this study that an effort be made to consider the fine‐scale variability of any feature of the environment that may impede detection of their target species.

## 5 CONCLUSION

This study demonstrates a novel, multifaceted approach to produce a dynamic occupancy model for a species of concern in a closed area. It has generated estimates of occupancy that are considerably greater than occupancy measured from observation alone, for the first time using outputs from a high‐resolution 3‐dimensional hydrodynamic model in such a modeling framework. While the need for species distribution models to consider the 3‐dimensional nature of the marine environment has been documented previously (Duffy & Chown, 2017), this study reinforces it using modern statistical methods. Using the outputs, this investigation has shown how the behavior of cusk changes in two different environments as a function of current speed. This behavior has implications for the detectability of the species, which in turn has implications for the occupancy estimates. This imperative to consider detectability in marine SDM studies is true not only for cusk, but for all species surveyed using noninvasive sampling methods. It holds especially true for areas where managers must use these methods to monitor stocks. Failing to recognize the limitations of models that do not account for imperfect detection will impact future estimates of abundance, potentially for many species. It is imperative that practitioners of future marine SDM applications consider the detectability of the species under study. In order to do this, they must first understand the processes that govern the fine scale, time‐varying features of the environment that may affect detectability, not just for the species in question but also for the specific habitat type being observed (Bacheler et al., 2014) in order to obtain unbiased estimates of occupancy in the marine environment.

## AUTHOR CONTRIBUTIONS

J.C., C.M. and B.H. conceived of and designed the investigation, J.G. collected data, S.A.S guided the analytical process, R.Q. advised on technical aspects of manuscript. All authors contributed to the preparation of final manuscript and gave approval for submission.

## DATA ACCESSIBILITY

Data will be archived on Dryad.
