# Supplementary material for: Dynamic occupancy modeling of temperate marine fish in area‐based closures
Source: Ecol Evol. 2018 Sep 21;8(20):10192–205. doi: 10.1002/ece3.4493 (PMC6206187; doi:10.1002/ece3.4493)
Supplement: Supplementary file 1 [file ECE3-8-10192-s001.docx]

**Mesh set up**

*Smallest allowable angle*: 28°.

*Maximum allowable area in deep water (>200 m)*: 0.01 degrees^2^.

*Maximum allowable area in shallow water outside study area*: 0.002 degrees^2^.

*Maximum allowable area inside study area*: 6e-6 degrees^2^.

*Number of steps to reduce mesh area*: 5, reducing area by one order of magnitude per step.

*Bathymetries inside study area*: 5 m MBES data (SAIC 2005).

*Bathymetries inside Gulf of Maine but not in study area*: USGS 3 arc second data (USGS 2013).

*Bathymetries outside Gulf of Maine*: TOPEX v17 30 arc second data (Ray 1999).

*Number of elements*: 27651

*Number of nodes*: 14779

*Number of terrain following sigma layers*: 10.

*Sigma depth*: 220 m.

*Number of equidistant z-levels*: 12.

*Depth of z-levels*: 220 m – 5065 m.

*Flood and dry depth*: 0.01 m.

**Model set up**

**Current model**

*Warm up period for each model run*: 30 days.

*Time step*: 1200 seconds.

*Density*: Barotropic.

*Eddy viscosity*: Smagorinsky formulation, constant value (0.28).

*Bed resistance*: Spatially varying based on USGS sediment map and empirical measurements of C_100_ (Johns 1983).

*Coriolis forcing*: spatially varying.

*Wind forcing*: Varying in time and domain. Modelled from NCEP climate forecast system reanalysis hourly data (Saha *et al.*, 2010; 2011).

*Wind friction*: Varying with wind speed.

*Wave radiation*: Included from wave simulation.

*Initial conditions*: constant 0.

*Boundary conditions*: Varying surface elevation in time along boundaries. Taken from the 0.125° DTU10 global ocean model (Andersen 2008; Cheng and Andersen 2010).

**Model set up**

**Wave model**

*Warm up period for each model run*: 30 days.

*Time step*: 1200 seconds.

*Formulation*: Fully spectral.

*Time formulation*: Instationary.

*Spectral discretisation*: logarithmic (25 frequencies, 0.055 Hz minimum, 1.1 freq. factor, 16 directions).

*Water level corrections*: From current simulation.

*Current conditions*: From current simulation.

*Wind forcing*: Varying in time and domain. Modelled from NCEP climate forecast system reanalysis hourly data (Saha *et al.*, 2010; 2011). Coupled air-sea.

*Diffraction*: Included, smoothing factor 1.

*Energy transfer*: Quadruplet wave action.

*Wave breaking*: Included, constant gamma (0.8). Alpha 1.

*Bottom friction*: Nikuradse roughness, constant (0.04 m).

*White capping*: Included. Dissipation coefficient constant (Cdis 4.5, DELTA dis 0.5).

*Initial conditions*: Spectra from empirical formulae. JONSWAP fetch growth expression defaults (max fetch 100000 m, max peak freq. 0.4 Hz, max Philips constant 0.0081).

*Boundary conditions*: All boundaries considered closed.

**Calibration**

The model was calibrated using a simulation for the year 2008. After each model run, harmonic analysis was conducted to calculate the amplitude and Greenwich phase lag of the M_2_, N_s_, S_2_, K_1_ and O_1_ tidal constituents at 63 locations in the Gulf of Maine (figure 1). These were compared against observed data taken from Moody *et al.* (1984). For model runs where the mean absolute error for any constituent was unacceptably high, adjustments were made to the quadratic drag coefficient to tune the model harmonics. Once the errors were at an acceptable standard, the model was deemed calibrated and set up to run for other years.


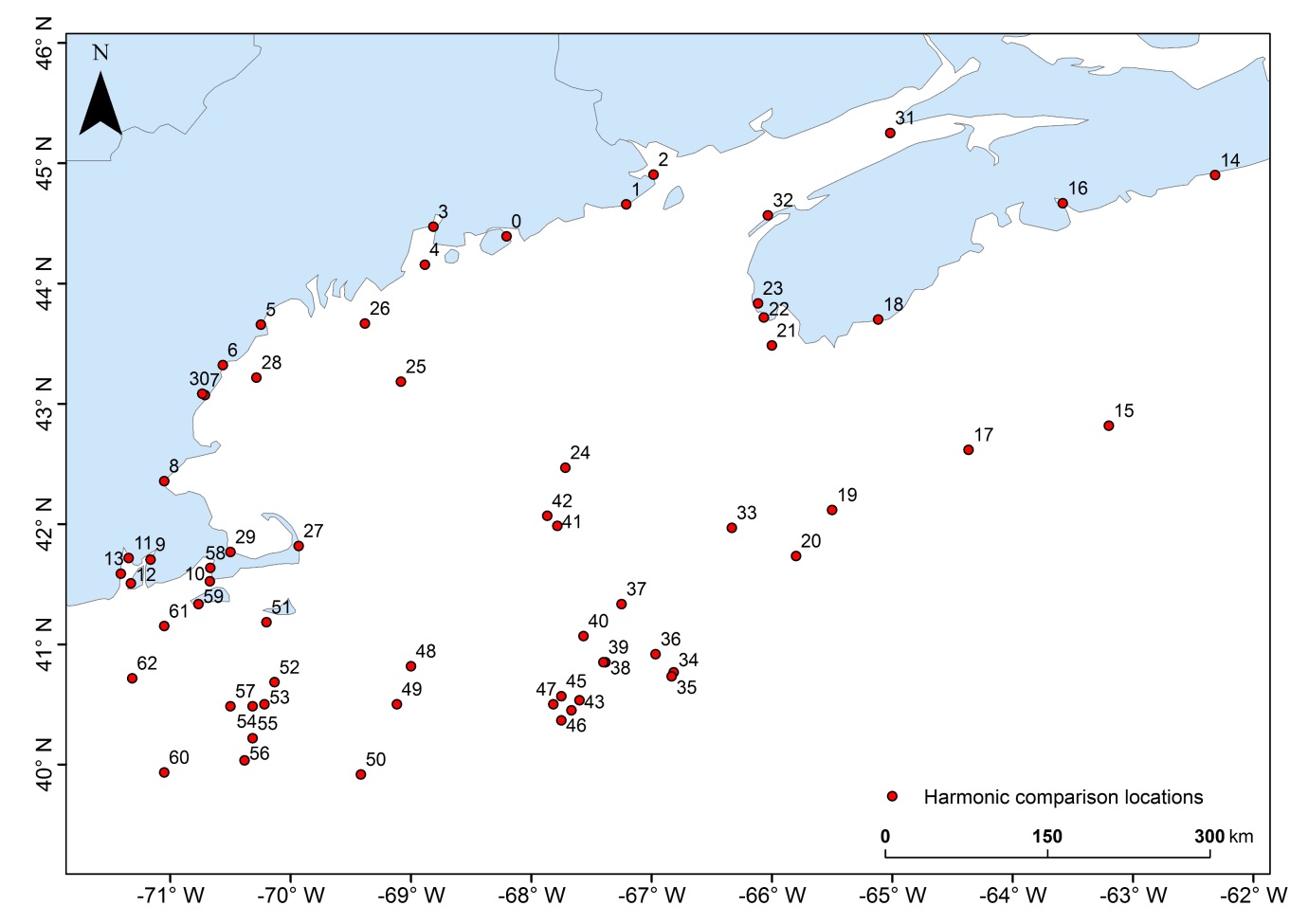
**Figure S1. Locations of 63 sites where harmonic analysis of the M_2_, N_2_, S_2_, K_1_ and O_1_ tidal constituents from the hydrodynamic model were compared to observed data. Numbers on the map correspond to row numbers in tables 2 - 6.**

The M_2_ tidal constituent dominates the Gulf of Maine Bay of Fundy system, giving rise to the large tidal ranges experienced in the region. Results of the harmonic analyses for the calibrated model show a mean absolute error in amplitude for the M_2_ of 3.7 cm, and mean absolute error in Greenwich phase lag of 5.27° (table 1 and table 2). Results for the other constituents are summarised in table 1 and given in full in tables 3 – 6.

Table S1. Mean absolute error and standard deviation of absolute error for amplitude (cm) and Greenwich phase lag (degrees) for 5 harmonic constituents at 63 locations in the Gulf of Maine.

|  | M_2_ Amp | M_2_ Phase | N_2_ Amp | N_2_ Phase | S_2_ Amp | S_2_ Phase | K_1_ Amp | K_1_ Phase | O_1_ Amp | O_1_ Phase |
| --- | --- | --- | --- | --- | --- | --- | --- | --- | --- | --- |
| Mean abs. err. | 3.7 | 5.3 | 1.5 | 6.4 | 1.8 | 6.3 | 1.1 | 8.2 | 1.5 | 8.0 |
| Std abs. err. | 4.6 | 4.6 | 2.0 | 5.9 | 3.9 | 6.0 | 0.7 | 6.8 | 1.0 | 6.6 |

Table S2. Observed, modelled and difference in amplitude and Greenwich phase lag for the M_2_ tidal constituent at 63 locations in the Gulf of Maine. Row numbers in column 1 correspond to the locations in figure 1.

| **Figure 1** | **Station** | **Amplitude** | **Modelled amp** | **Difference** | **Phase** | **Modelled phase** | **Difference** |
| --- | --- | --- | --- | --- | --- | --- | --- |
|  |  | **(m)** | **(m)** | **(m)** | **(° G)** | **(° G)** | **(° G)** |
| 0 | Bar Harbor | 1.58 | 1.58 | 0.00 | 92.9 | 85.6 | 7.35 |
| 1 | Cultler Farris Wharf | 2.034 | 2.01 | 0.02 | 93.4 | 87.2 | 6.17 |
| 2 | Eastport | 2.687 | 2.56 | 0.13 | 98.7 | 97.4 | 1.30 |
| 3 | Fort Point ME | 1.589 | 1.63 | 0.04 | 98.2 | 90.4 | 7.83 |
| 4 | Pulpit Harbor | 1.494 | 1.54 | 0.04 | 98.2 | 88.7 | 9.50 |
| 5 | Portland | 1.365 | 1.39 | 0.03 | 102.5 | 94.0 | 8.51 |
| 6 | Wells | 1.33 | 1.35 | 0.02 | 105.3 | 95.0 | 10.28 |
| 7 | Fort Point NH | 1.314 | 1.34 | 0.03 | 105.9 | 96.6 | 9.34 |
| 8 | Boston | 1.398 | 1.40 | 0.00 | 109.4 | 102.0 | 7.36 |
| 9 | Fall River | 0.614 | 0.55 | 0.06 | 8.7 | 357.6 | 11.12 |
| 10 | Woods Hole | 0.243 | 0.37 | 0.13 | 34.6 | 7.1 | 27.48 |
| 11 | Conimicut Light | 0.593 | 0.55 | 0.05 | 7.2 | 358.2 | 8.97 |
| 12 | Newport | 0.505 | 0.50 | 0.01 | 2.3 | 354.7 | 7.60 |
| 13 | Quonset Point | 0.538 | 0.52 | 0.02 | 5 | 357.9 | 7.13 |
| 14 | West Newdy | 0.607 | 0.61 | 0.00 | 347 | 346.6 | 0.45 |
| 15 | B1_ta | 0.482 | 0.48 | 0.00 | 351 | 349.6 | 1.38 |
| 16 | Halifax | 0.63 | 0.59 | 0.04 | 349 | 1.3 | 12.25 |
| 17 | T21_ta | 0.49 | 0.49 | 0.00 | 357 | 354.4 | 2.55 |
| 18 | Lockport | 0.698 | 0.75 | 0.05 | 359 | 356.8 | 2.20 |
| 19 | T22A_ta | 0.458 | 0.48 | 0.02 | 4 | 4.1 | 0.07 |
| 20 | T3_ta | 0.396 | 0.42 | 0.02 | 2 | 2.5 | 0.49 |
| 21 | Seal Island | 1.204 | 1.36 | 0.16 | 52 | 43.3 | 8.73 |
| 22 | Pickney | 1.554 | 1.59 | 0.03 | 59 | 52.0 | 7.01 |
| 23 | Yarmouth | 1.632 | 1.46 | 0.17 | 63 | 59.5 | 3.50 |
| 24 | B6_ta | 0.88 | 0.92 | 0.04 | 87 | 79.3 | 7.72 |
| 25 | Cashes Ledge | 1.2 | 1.26 | 0.06 | 98 | 89.4 | 8.60 |
| 26 | Monhegan | 1.3 | 1.37 | 0.07 | 99 | 90.4 | 8.61 |
| 27 | Nauset | 1.03 | 1.04 | 0.01 | 102 | 106.5 | 4.51 |
| 28 | C. Porpoise | 1.27 | 1.33 | 0.06 | 103 | 94.7 | 8.29 |
| 29 | Cape Cod Canl | 1.244 | 1.41 | 0.17 | 109 | 102.3 | 6.71 |
| 30 | Portsmouth | 1.303 | 1.35 | 0.05 | 107 | 96.7 | 10.30 |
| 31 | Isle Haute | 4.18 | 4.23 | 0.05 | 98 | 96.6 | 1.41 |
| 32 | Centreville | 2.606 | 2.78 | 0.17 | 92 | 80.3 | 11.71 |
| 33 | M7_ta | 0.41 | 0.44 | 0.03 | 38 | 38.4 | 0.42 |
| 34 | M5_ta | 0.405 | 0.40 | 0.00 | 356 | 356.5 | 0.45 |
| 35 | T4_ta | 0.404 | 0.40 | 0.00 | 355 | 356.0 | 0.99 |
| 36 | M4_ta | 0.389 | 0.40 | 0.01 | 1 | 2.4 | 1.39 |
| 37 | M3_ta | 0.396 | 0.40 | 0.00 | 22 | 30.4 | 8.43 |
| 38 | M9_ta | 0.389 | 0.40 | 0.01 | 6 | 7.1 | 1.15 |
| 39 | A_ta | 0.389 | 0.40 | 0.01 | 5 | 7.4 | 2.44 |
| 40 | K_ta | 0.399 | 0.41 | 0.01 | 18 | 22.4 | 4.43 |
| 41 | D_ta | 0.758 | 0.81 | 0.05 | 93 | 84.5 | 8.50 |
| 42 | M1_ta | 0.782 | 0.84 | 0.06 | 92 | 84.2 | 7.78 |
| 43 | LCL_ta | 0.394 | 0.40 | 0.01 | 354 | 358.4 | 4.42 |
| 44 | LC0_ta | 0.392 | 0.40 | 0.01 | 357 | 356.7 | 0.29 |
| 45 | LCA_ta | 0.392 | 0.40 | 0.01 | 358 | 0.9 | 2.85 |
| 46 | T23_ta | 0.407 | 0.41 | 0.00 | 356 | 355.1 | 0.86 |
| 47 | LCM_ta | 0.394 | 0.40 | 0.00 | 356 | 359.2 | 3.20 |
| 48 | B_ta | 0.259 | 0.29 | 0.03 | 47 | 47.4 | 0.41 |
| 49 | R_ta | 0.314 | 0.32 | 0.01 | 3 | 8.4 | 5.40 |
| 50 | Kiwi_ta | 0.414 | 0.41 | 0.00 | 349 | 351.3 | 2.27 |
| 51 | S_ta | 0.323 | 0.29 | 0.04 | 1 | 359.4 | 1.61 |
| 52 | NSFE1_ta | 0.387 | 0.36 | 0.02 | 356 | 356.1 | 0.15 |
| 53 | Q_ta | 0.387 | 0.39 | 0.00 | 353 | 353.5 | 0.47 |
| 54 | NSFE2_ta | 0.404 | 0.40 | 0.01 | 354 | 352.8 | 1.19 |
| 55 | NSFE4_ta | 0.42 | 0.41 | 0.01 | 353 | 351.2 | 1.84 |
| 56 | NSFE5_ta | 0.419 | 0.42 | 0.00 | 351 | 350.4 | 0.62 |
| 57 | P_ta | 0.416 | 0.41 | 0.01 | 352 | 352.1 | 0.13 |
| 58 | BBA_ta | 0.538 | 0.42 | 0.12 | 8 | 3.1 | 4.89 |
| 59 | Menemsha | 0.451 | 0.39 | 0.07 | 5 | 348.5 | 16.55 |
| 60 | NES763_ta | 0.433 | 0.42 | 0.01 | 349 | 349.7 | 0.67 |
| 61 | CXL_ta | 0.444 | 0.44 | 0.00 | 1 | 352.1 | 8.86 |
| 62 | Picket | 0.44 | 0.44 | 0.00 | 349 | 351.8 | 2.84 |

Table S3. Observed, modelled and difference in amplitude and Greenwich phase lag for the N_2_ tidal constituent at 63 locations in the Gulf of Maine. Row numbers in column 1 correspond to the locations in figure 1.

| **Figure 1** | **Station** | **Amplitude** | **Modelled amp** | **Difference** | **Phase** | **Modelled phase** | **Difference** |
| --- | --- | --- | --- | --- | --- | --- | --- |
|  |  | **(m)** | **(m)** | | **(m)** | **(° G)** | **(° G)** |
| 0 | Bar Harbor | 0.351 | 0.359 | 0.01 | 62.3 | 53.7 | 8.59 |
| 1 | Cultler Farris Wharf | 0.446 | 0.442 | 0.00 | 63.3 | 55.3 | 8.03 |
| 2 | Eastport | 0.543 | 0.542 | 0.00 | 69.4 | 65.9 | 3.50 |
| 3 | Fort Point ME | 0.355 | 0.373 | 0.02 | 63.5 | 59.4 | 4.14 |
| 4 | Pulpit Harbor | 0.342 | 0.353 | 0.01 | 65.9 | 57.3 | 8.64 |
| 5 | Portland | 0.306 | 0.323 | 0.02 | 72 | 62.3 | 9.73 |
| 6 | Wells | 0.294 | 0.314 | 0.02 | 71.4 | 63.2 | 8.17 |
| 7 | Fort Point NH | 0.294 | 0.310 | 0.02 | 76.1 | 65.1 | 11.00 |
| 8 | Boston | 0.309 | 0.324 | 0.02 | 78.9 | 71.9 | 6.99 |
| 9 | Fall River | 0.151 | 0.137 | 0.01 | 353.5 | 342.0 | 11.53 |
| 10 | Woods Hole | 0.08 | 0.107 | 0.03 | 19.4 | 351.2 | 28.15 |
| 11 | Conimicut Light | 0.147 | 0.135 | 0.01 | 351.9 | 342.4 | 9.48 |
| 12 | Newport | 0.124 | 0.124 | 0.00 | 345.8 | 338.0 | 7.76 |
| 13 | Quonset Point | 0.133 | 0.130 | 0.00 | 349.4 | 341.7 | 7.66 |
| 14 | West Newdy | 0.169 | 0.135 | 0.03 | 328 | 325.8 | 2.22 |
| 15 | B1_ta | 0.119 | 0.111 | 0.01 | 329 | 329.0 | 0.05 |
| 16 | Halifax | 0.143 | 0.125 | 0.02 | 329 | 343.8 | 14.75 |
| 17 | T21_ta | 0.116 | 0.115 | 0.00 | 335 | 332.7 | 2.34 |
| 18 | Lockport | 0.171 | 0.167 | 0.00 | 337 | 332.3 | 4.66 |
| 19 | T22A_ta | 0.107 | 0.114 | 0.01 | 343 | 340.8 | 2.22 |
| 20 | T3_ta | 0.103 | 0.101 | 0.00 | 341 | 339.8 | 1.18 |
| 21 | Seal Island | 0.253 | 0.305 | 0.05 | 24 | 14.4 | 9.64 |
| 22 | Pickney | 0.299 | 0.335 | 0.04 | 49 | 22.3 | 26.65 |
| 23 | Yarmouth | 0.349 | 0.276 | 0.07 | 33 | 28.5 | 4.48 |
| 24 | B6_ta | 0.216 | 0.216 | 0.00 | 57 | 49.3 | 7.70 |
| 25 | Cashes Ledge | 0.282 | 0.293 | 0.01 | 66 | 58.1 | 7.91 |
| 26 | Monhegan | 0.303 | 0.317 | 0.01 | 67 | 58.8 | 8.21 |
| 27 | Nauset | 0.222 | 0.246 | 0.02 | 70 | 74.3 | 4.26 |
| 28 | C. Porpoise | 0.299 | 0.309 | 0.01 | 71 | 63.0 | 8.01 |
| 29 | Cape Cod Canl | 0.289 | 0.328 | 0.04 | 74 | 70.6 | 3.37 |
| 30 | Portsmouth | 0.278 | 0.313 | 0.03 | 76 | 65.1 | 10.94 |
| 31 | Isle Haute | 0.866 | 0.868 | 0.00 | 74 | 63.9 | 10.05 |
| 32 | Centreville | 0.728 | 0.592 | 0.14 | 67 | 48.7 | 18.35 |
| 33 | M7_ta | 0.097 | 0.109 | 0.01 | 12 | 11.0 | 1.00 |
| 34 | M5_ta | 0.093 | 0.100 | 0.01 | 337 | 333.6 | 3.37 |
| 35 | T4_ta | 0.105 | 0.100 | 0.00 | 337 | 333.2 | 3.78 |
| 36 | M4_ta | 0.101 | 0.101 | 0.00 | 349 | 338.6 | 10.36 |
| 37 | M3_ta | 0.1 | 0.102 | 0.00 | 354 | 1.9 | 7.91 |
| 38 | M9_ta | 0.107 | 0.105 | 0.00 | 346 | 342.6 | 3.44 |
| 39 | A_ta | 0.096 | 0.105 | 0.01 | 344 | 342.8 | 1.20 |
| 40 | K_ta | 0.099 | 0.109 | 0.01 | 354 | 355.0 | 1.01 |
| 41 | D_ta | 0.186 | 0.193 | 0.01 | 63 | 55.1 | 7.88 |
| 42 | M1_ta | 0.18 | 0.200 | 0.02 | 63 | 54.7 | 8.34 |
| 43 | LCL_ta | 0.091 | 0.104 | 0.01 | 330 | 335.6 | 5.59 |
| 44 | LC0_ta | 0.093 | 0.104 | 0.01 | 333 | 334.2 | 1.23 |
| 45 | LCA_ta | 0.098 | 0.105 | 0.01 | 338 | 337.8 | 0.20 |
| 46 | T23_ta | 0.099 | 0.105 | 0.01 | 336 | 332.9 | 3.07 |
| 47 | LCM_ta | 0.095 | 0.104 | 0.01 | 334 | 336.5 | 2.53 |
| 48 | B_ta | 0.075 | 0.084 | 0.01 | 20 | 19.4 | 0.61 |
| 49 | R_ta | 0.079 | 0.091 | 0.01 | 344 | 347.9 | 3.94 |
| 50 | Kiwi_ta | 0.11 | 0.105 | 0.00 | 334 | 332.4 | 1.60 |
| 51 | S_ta | 0.091 | 0.083 | 0.01 | 339 | 346.0 | 7.02 |
| 52 | NSFE1_ta | 0.094 | 0.097 | 0.00 | 340 | 339.1 | 0.94 |
| 53 | Q_ta | 0.093 | 0.100 | 0.01 | 337 | 336.3 | 0.67 |
| 54 | NSFE2_ta | 0.096 | 0.102 | 0.01 | 338 | 335.7 | 2.35 |
| 55 | NSFE4_ta | 0.097 | 0.103 | 0.01 | 336 | 333.8 | 2.21 |
| 56 | NSFE5_ta | 0.103 | 0.104 | 0.00 | 335 | 332.7 | 2.27 |
| 57 | P_ta | 0.103 | 0.103 | 0.00 | 337 | 334.9 | 2.06 |
| 58 | BBA_ta | 0.138 | 0.116 | 0.02 | 351 | 347.4 | 3.60 |
| 59 | Menemsha | 0.119 | 0.099 | 0.02 | 356 | 333.8 | 22.22 |
| 60 | NES763_ta | 0.104 | 0.102 | 0.00 | 332 | 331.8 | 0.19 |
| 61 | CXL_ta | 0.103 | 0.113 | 0.01 | 334 | 334.8 | 0.80 |
| 62 | Picket | 0.12 | 0.111 | 0.01 | 317 | 333.7 | 16.68 |

Table S4. Observed, modelled and difference in amplitude and Greenwich phase lag for the S_2_ tidal constituent at 63 locations in the Gulf of Maine. Row numbers in column 1 correspond to the locations in figure 1.

| **Figure 1** | **Station** | **Amplitude** | **Modelled amp** | **Difference** | **Phase** | **Modelled phase** | **Difference** |
| --- | --- | --- | --- | --- | --- | --- | --- |
|  |  | **(m)** | **(m)** | **(m)** | **(° G)** | **(° G)** | **(° G)** |
| 0 | Bar Harbor | 0.243 | 0.260 | 0.02 | 128.8 | 121.9 | 6.94 |
| 1 | Cultler Farris Wharf | 0.309 | 0.334 | 0.02 | 131 | 124.7 | 6.31 |
| 2 | Eastport | 0.42 | 0.421 | 0.00 | 139.3 | 137.0 | 2.29 |
| 3 | Fort Point ME | 0.227 | 0.268 | 0.04 | 134.5 | 127.5 | 6.96 |
| 4 | Pulpit Harbor | 0.218 | 0.253 | 0.03 | 134.1 | 125.2 | 8.88 |
| 5 | Portland | 0.206 | 0.227 | 0.02 | 138.5 | 130.7 | 7.78 |
| 6 | Wells | 0.203 | 0.221 | 0.02 | 141.7 | 131.8 | 9.87 |
| 7 | Fort Point NH | 0.181 | 0.218 | 0.04 | 136.2 | 133.9 | 2.25 |
| 8 | Boston | 0.213 | 0.230 | 0.02 | 146.2 | 141.5 | 4.74 |
| 9 | Fall River | 0.131 | 0.124 | 0.01 | 31.1 | 20.9 | 10.18 |
| 10 | Woods Hole | 0.061 | 0.088 | 0.03 | 35.6 | 22.5 | 13.10 |
| 11 | Conimicut Light | 0.13 | 0.122 | 0.01 | 30 | 21.4 | 8.58 |
| 12 | Newport | 0.108 | 0.112 | 0.00 | 25 | 17.0 | 7.96 |
| 13 | Quonset Point | 0.116 | 0.117 | 0.00 | 27.1 | 20.9 | 6.24 |
| 14 | West Newdy | 0.166 | 0.135 | 0.03 | 26 | 20.2 | 5.83 |
| 15 | B1_ta | 0.103 | 0.104 | 0.00 | 22 | 18.4 | 3.64 |
| 16 | Halifax | 0.143 | 0.119 | 0.02 | 21 | 40.0 | 18.96 |
| 17 | T21_ta | 0.103 | 0.103 | 0.00 | 23 | 22.1 | 0.92 |
| 18 | Lockport | 0.137 | 0.147 | 0.01 | 29 | 28.8 | 0.22 |
| 19 | T22A_ta | 0.094 | 0.096 | 0.00 | 28 | 29.1 | 1.08 |
| 20 | T3_ta | 0.082 | 0.086 | 0.00 | 28 | 26.1 | 1.89 |
| 21 | Seal Island | 0.21 | 0.232 | 0.02 | 86 | 77.4 | 8.63 |
| 22 | Pickney | 0.235 | 0.256 | 0.02 | 92 | 87.7 | 4.31 |
| 23 | Yarmouth | 0.269 | 0.210 | 0.06 | 97 | 96.0 | 0.98 |
| 24 | B6_ta | 0.129 | 0.146 | 0.02 | 119 | 113.0 | 5.98 |
| 25 | Cashes Ledge | 0.195 | 0.205 | 0.01 | 126 | 125.4 | 0.63 |
| 26 | Monhegan | 0.211 | 0.223 | 0.01 | 128 | 126.7 | 1.34 |
| 27 | Nauset | 0.144 | 0.166 | 0.02 | 133 | 143.5 | 10.54 |
| 28 | C. Porpoise | 0.203 | 0.217 | 0.01 | 134 | 131.4 | 2.62 |
| 29 | Cape Cod Canl | 0.199 | 0.232 | 0.03 | 144 | 139.5 | 4.50 |
| 30 | Portsmouth | 0.203 | 0.220 | 0.02 | 143 | 133.9 | 9.13 |
| 31 | Isle Haute | 0.422 | 0.711 | 0.29 | 159 | 136.2 | 22.82 |
| 32 | Centreville | 0.424 | 0.468 | 0.04 | 137 | 118.5 | 18.55 |
| 33 | M7_ta | 0.086 | 0.077 | 0.01 | 59 | 59.4 | 0.35 |
| 34 | M5_ta | 0.092 | 0.087 | 0.01 | 24 | 19.0 | 4.96 |
| 35 | T4_ta | 0.084 | 0.087 | 0.00 | 24 | 18.6 | 5.36 |
| 36 | M4_ta | 0.084 | 0.084 | 0.00 | 26 | 23.4 | 2.63 |
| 37 | M3_ta | 0.098 | 0.073 | 0.03 | 15 | 46.9 | 31.90 |
| 38 | M9_ta | 0.079 | 0.084 | 0.01 | 32 | 26.3 | 5.67 |
| 39 | A_ta | 0.087 | 0.084 | 0.00 | 27 | 26.5 | 0.47 |
| 40 | K_ta | 0.086 | 0.080 | 0.01 | 38 | 39.3 | 1.31 |
| 41 | D_ta | 0.111 | 0.127 | 0.02 | 120 | 118.4 | 1.62 |
| 42 | M1_ta | 0.122 | 0.133 | 0.01 | 121 | 118.3 | 2.72 |
| 43 | LCL_ta | 0.095 | 0.088 | 0.01 | 19 | 19.0 | 0.00 |
| 44 | LC0_ta | 0.091 | 0.089 | 0.00 | 21 | 17.6 | 3.35 |
| 45 | LCA_ta | 0.088 | 0.087 | 0.00 | 21 | 20.6 | 0.37 |
| 46 | T23_ta | 0.086 | 0.090 | 0.00 | 20 | 16.4 | 3.60 |
| 47 | LCM_ta | 0.092 | 0.088 | 0.00 | 21 | 19.2 | 1.78 |
| 48 | B_ta | 0.05 | 0.047 | 0.00 | 59 | 54.9 | 4.14 |
| 49 | R_ta | 0.076 | 0.072 | 0.00 | 21 | 23.9 | 2.89 |
| 50 | Kiwi_ta | 0.081 | 0.091 | 0.01 | 15 | 14.3 | 0.67 |
| 51 | S_ta | 0.078 | 0.069 | 0.01 | 21 | 15.9 | 5.13 |
| 52 | NSFE1_ta | 0.089 | 0.081 | 0.01 | 18 | 16.1 | 1.92 |
| 53 | Q_ta | 0.087 | 0.085 | 0.00 | 18 | 15.1 | 2.94 |
| 54 | NSFE2_ta | 0.087 | 0.086 | 0.00 | 17 | 14.7 | 2.26 |
| 55 | NSFE4_ta | 0.092 | 0.088 | 0.00 | 18 | 14.4 | 3.62 |
| 56 | NSFE5_ta | 0.091 | 0.089 | 0.00 | 17 | 14.3 | 2.74 |
| 57 | P_ta | 0.095 | 0.088 | 0.01 | 15 | 14.3 | 0.67 |
| 58 | BBA_ta | 0.116 | 0.098 | 0.02 | 32 | 20.7 | 11.30 |
| 59 | Menemsha | 0.1 | 0.088 | 0.01 | 24 | 9.5 | 14.46 |
| 60 | NES763_ta | 0.089 | 0.086 | 0.00 | 17 | 13.5 | 3.47 |
| 61 | CXL_ta | 0.099 | 0.098 | 0.00 | 0 | 13.4 | 13.35 |
| 62 | Picket | 0.095 | 0.094 | 0.00 | 0 | 13.4 | 13.40 |

Table S5. Observed, modelled and difference in amplitude and Greenwich phase lag for the K_1_ tidal constituent at 63 locations in the Gulf of Maine. Row numbers in column 1 correspond to the locations in figure 1.

| **Figure 1** | **Station** | **Amplitude** | **Modelled amp** | **Difference** | **Phase** | **Modelled phase** | **Difference** |
| --- | --- | --- | --- | --- | --- | --- | --- |
|  |  | **(m)** | **(m)** | **(m)** | **(° G)** | **(° G)** | **(° G)** |
| 0 | Bar Harbor | 0.14 | 0.122 | 0.02 | 194.3 | 197.4 | 3.11 |
| 1 | Cultler Farris Wharf | 0.149 | 0.126 | 0.02 | 191.9 | 195.6 | 3.72 |
| 2 | Eastport | 0.156 | 0.133 | 0.02 | 196.4 | 199.9 | 3.54 |
| 3 | Fort Point ME | 0.148 | 0.127 | 0.02 | 195.9 | 201.8 | 5.94 |
| 4 | Pulpit Harbor | 0.141 | 0.124 | 0.02 | 197.7 | 201.2 | 3.46 |
| 5 | Portland | 0.141 | 0.123 | 0.02 | 202.2 | 206.1 | 3.89 |
| 6 | Wells | 0.137 | 0.122 | 0.01 | 203.7 | 207.0 | 3.34 |
| 7 | Fort Point NH | 0.135 | 0.122 | 0.01 | 203.3 | 208.5 | 5.20 |
| 8 | Boston | 0.143 | 0.123 | 0.02 | 205.2 | 211.7 | 6.49 |
| 9 | Fall River | 0.07 | 0.059 | 0.01 | 168.9 | 148.6 | 20.28 |
| 10 | Woods Hole | 0.071 | 0.049 | 0.02 | 189 | 161.5 | 27.49 |
| 11 | Conimicut Light | 0.068 | 0.059 | 0.01 | 165.7 | 149.1 | 16.61 |
| 12 | Newport | 0.062 | 0.056 | 0.01 | 166.1 | 147.7 | 18.37 |
| 13 | Quonset Point | 0.064 | 0.057 | 0.01 | 165.1 | 149.1 | 15.97 |
| 14 | West Newdy | 0.088 | 0.093 | 0.01 | 95 | 107.0 | 11.95 |
| 15 | B1_ta | 0.069 | 0.067 | 0.00 | 173 | 172.3 | 0.72 |
| 16 | Halifax | 0.103 | 0.101 | 0.00 | 121 | 139.0 | 18.02 |
| 17 | T21_ta | 0.068 | 0.069 | 0.00 | 170 | 166.8 | 3.21 |
| 18 | Lockport | 0.128 | 0.128 | 0.00 | 147 | 154.9 | 7.92 |
| 19 | T22A_ta | 0.071 | 0.080 | 0.01 | 167 | 168.5 | 1.49 |
| 20 | T3_ta | 0.071 | 0.079 | 0.01 | 170 | 172.2 | 2.21 |
| 21 | Seal Island | 0.137 | 0.120 | 0.02 | 179 | 179.3 | 0.30 |
| 22 | Pickney | 0.122 | 0.116 | 0.01 | 184 | 186.0 | 2.05 |
| 23 | Yarmouth | 0.135 | 0.098 | 0.04 | 182 | 199.2 | 17.24 |
| 24 | B6_ta | 0.108 | 0.095 | 0.01 | 196 | 198.7 | 2.72 |
| 25 | Cashes Ledge | 0.125 | 0.113 | 0.01 | 198 | 203.4 | 5.40 |
| 26 | Monhegan | 0.136 | 0.120 | 0.02 | 194 | 203.5 | 9.46 |
| 27 | Nauset | 0.131 | 0.114 | 0.02 | 201 | 213.5 | 12.46 |
| 28 | C. Porpoise | 0.129 | 0.120 | 0.01 | 204 | 206.9 | 2.90 |
| 29 | Cape Cod Canl | 0.131 | 0.122 | 0.01 | 206 | 211.8 | 5.82 |
| 30 | Portsmouth | 0.141 | 0.122 | 0.02 | 204 | 208.2 | 4.23 |
| 31 | Isle Haute | 0.165 | 0.146 | 0.02 | 196 | 193.9 | 2.14 |
| 32 | Centreville | 0.149 | 0.133 | 0.02 | 189 | 189.8 | 0.76 |
| 33 | M7_ta | 0.076 | 0.076 | 0.00 | 182 | 179.8 | 2.21 |
| 34 | M5_ta | 0.08 | 0.077 | 0.00 | 171 | 178.7 | 7.69 |
| 35 | T4_ta | 0.077 | 0.077 | 0.00 | 172 | 178.7 | 6.65 |
| 36 | M4_ta | 0.075 | 0.077 | 0.00 | 168 | 180.0 | 11.97 |
| 37 | M3_ta | 0.066 | 0.072 | 0.01 | 178 | 185.4 | 7.36 |
| 38 | M9_ta | 0.072 | 0.074 | 0.00 | 170 | 181.5 | 11.50 |
| 39 | A_ta | 0.076 | 0.073 | 0.00 | 173 | 181.6 | 8.56 |
| 40 | K_ta | 0.074 | 0.073 | 0.00 | 176 | 184.3 | 8.31 |
| 41 | D_ta | 0.102 | 0.091 | 0.01 | 198 | 201.9 | 3.90 |
| 42 | M1_ta | 0.112 | 0.092 | 0.02 | 199 | 202.0 | 2.98 |
| 43 | LCL_ta | 0.08 | 0.072 | 0.01 | 171 | 179.3 | 8.35 |
| 44 | LC0_ta | 0.084 | 0.072 | 0.01 | 173 | 178.8 | 5.82 |
| 45 | LCA_ta | 0.078 | 0.072 | 0.01 | 169 | 179.7 | 10.71 |
| 46 | T23_ta | 0.079 | 0.072 | 0.01 | 172 | 178.4 | 6.41 |
| 47 | LCM_ta | 0.08 | 0.072 | 0.01 | 172 | 179.4 | 7.36 |
| 48 | B_ta | 0.073 | 0.062 | 0.01 | 196 | 199.9 | 3.87 |
| 49 | R_ta | 0.073 | 0.060 | 0.01 | 178 | 182.9 | 4.90 |
| 50 | Kiwi_ta | 0.087 | 0.072 | 0.01 | 176 | 171.4 | 4.64 |
| 51 | S_ta | 0.061 | 0.036 | 0.02 | 177 | 186.1 | 9.11 |
| 52 | NSFE1_ta | 0.065 | 0.052 | 0.01 | 173 | 167.1 | 5.95 |
| 53 | Q_ta | 0.073 | 0.062 | 0.01 | 173 | 167.5 | 5.47 |
| 54 | NSFE2_ta | 0.073 | 0.065 | 0.01 | 173 | 167.4 | 5.56 |
| 55 | NSFE4_ta | 0.081 | 0.073 | 0.01 | 177 | 168.6 | 8.37 |
| 56 | NSFE5_ta | 0.086 | 0.078 | 0.01 | 175 | 169.2 | 5.83 |
| 57 | P_ta | 0.083 | 0.068 | 0.01 | 177 | 167.4 | 9.62 |
| 58 | BBA_ta | 0.066 | 0.050 | 0.02 | 168 | 157.2 | 10.78 |
| 59 | Menemsha | 0.054 | 0.034 | 0.02 | 176 | 160.7 | 15.26 |
| 60 | NES763_ta | 0.087 | 0.084 | 0.00 | 178 | 171.6 | 6.37 |
| 61 | CXL_ta | 0.065 | 0.056 | 0.01 | 178 | 156.4 | 21.61 |
| 62 | Picket | 0.079 | 0.072 | 0.01 | 167 | 167.1 | 0.12 |

Table S6. Observed, modelled and difference in amplitude and Greenwich phase lag for the O_1_ tidal constituent at 63 locations in the Gulf of Maine. Row numbers in column 1 correspond to the locations in figure 1.

| **Figure 1** | **Station** | **Amplitude** | **Modelled amp** | **Difference** | **Phase** | **Modelled phase** | **Difference** |
| --- | --- | --- | --- | --- | --- | --- | --- |
|  |  | **(m)** | **(m)** | **(m)** | **(° G)** | **(° G)** | **(° G)** |
| 0 | Bar Harbor | 0.11 | 0.090 | 0.02 | 176.1 | 176.9 | 0.77 |
| 1 | Cultler Farris Wharf | 0.108 | 0.094 | 0.01 | 170.7 | 176.6 | 5.88 |
| 2 | Eastport | 0.119 | 0.099 | 0.02 | 176.9 | 180.9 | 4.04 |
| 3 | Fort Point ME | 0.114 | 0.093 | 0.02 | 177.9 | 180.2 | 2.30 |
| 4 | Pulpit Harbor | 0.111 | 0.091 | 0.02 | 178.6 | 179.3 | 0.72 |
| 5 | Portland | 0.112 | 0.088 | 0.02 | 182.4 | 182.2 | 0.23 |
| 6 | Wells | 0.111 | 0.087 | 0.02 | 186.5 | 182.6 | 3.87 |
| 7 | Fort Point NH | 0.114 | 0.087 | 0.03 | 187.1 | 183.9 | 3.17 |
| 8 | Boston | 0.119 | 0.089 | 0.03 | 186.7 | 186.5 | 0.16 |
| 9 | Fall River | 0.052 | 0.082 | 0.03 | 200.3 | 200.8 | 0.52 |
| 10 | Woods Hole | 0.066 | 0.082 | 0.02 | 203.7 | 194.0 | 9.68 |
| 11 | Conimicut Light | 0.051 | 0.082 | 0.03 | 198.6 | 201.0 | 2.44 |
| 12 | Newport | 0.047 | 0.080 | 0.03 | 202 | 199.9 | 2.05 |
| 13 | Quonset Point | 0.048 | 0.081 | 0.03 | 198.2 | 201.3 | 3.09 |
| 14 | West Newdy | 0.044 | 0.041 | 0.00 | 57 | 71.9 | 14.89 |
| 15 | B1_ta | 0.057 | 0.052 | 0.00 | 170 | 179.6 | 9.55 |
| 16 | Halifax | 0.048 | 0.056 | 0.01 | 93 | 119.8 | 26.83 |
| 17 | T21_ta | 0.054 | 0.047 | 0.01 | 179 | 173.3 | 5.67 |
| 18 | Lockport | 0.076 | 0.087 | 0.01 | 116 | 131.9 | 15.89 |
| 19 | T22A_ta | 0.055 | 0.049 | 0.01 | 179 | 165.1 | 13.87 |
| 20 | T3_ta | 0.058 | 0.051 | 0.01 | 179 | 167.1 | 11.93 |
| 21 | Seal Island | 0.104 | 0.091 | 0.01 | 163 | 161.2 | 1.83 |
| 22 | Pickney | 0.104 | 0.090 | 0.01 | 169 | 169.7 | 0.69 |
| 23 | Yarmouth | 0.101 | 0.084 | 0.02 | 164 | 187.1 | 23.06 |
| 24 | B6_ta | 0.092 | 0.075 | 0.02 | 181 | 179.8 | 1.20 |
| 25 | Cashes Ledge | 0.101 | 0.084 | 0.02 | 186 | 180.9 | 5.12 |
| 26 | Monhegan | 0.103 | 0.087 | 0.02 | 181 | 180.7 | 0.34 |
| 27 | Nauset | 0.115 | 0.083 | 0.03 | 182 | 188.2 | 6.24 |
| 28 | C. Porpoise | 0.106 | 0.087 | 0.02 | 185 | 182.7 | 2.34 |
| 29 | Cape Cod Canl | 0.108 | 0.088 | 0.02 | 187 | 186.9 | 0.14 |
| 30 | Portsmouth | 0.112 | 0.087 | 0.02 | 185 | 183.5 | 1.54 |
| 31 | Isle Haute | 0.119 | 0.109 | 0.01 | 158 | 178.1 | 20.06 |
| 32 | Centreville | 0.116 | 0.100 | 0.02 | 171 | 172.4 | 1.42 |
| 33 | M7_ta | 0.065 | 0.054 | 0.01 | 178 | 173.1 | 4.87 |
| 34 | M5_ta | 0.061 | 0.056 | 0.00 | 178 | 165.7 | 12.25 |
| 35 | T4_ta | 0.06 | 0.057 | 0.00 | 177 | 165.8 | 11.22 |
| 36 | M4_ta | 0.058 | 0.055 | 0.00 | 185 | 165.8 | 19.18 |
| 37 | M3_ta | 0.066 | 0.053 | 0.01 | 179 | 174.4 | 4.63 |
| 38 | M9_ta | 0.056 | 0.057 | 0.00 | 188 | 165.7 | 22.26 |
| 39 | A_ta | 0.061 | 0.057 | 0.00 | 179 | 165.7 | 13.26 |
| 40 | K_ta | 0.062 | 0.055 | 0.01 | 180 | 168.1 | 11.91 |
| 41 | D_ta | 0.085 | 0.074 | 0.01 | 184 | 182.3 | 1.69 |
| 42 | M1_ta | 0.085 | 0.074 | 0.01 | 185 | 182.2 | 2.84 |
| 43 | LCL_ta | 0.061 | 0.062 | 0.00 | 180 | 166.0 | 13.99 |
| 44 | LC0_ta | 0.065 | 0.063 | 0.00 | 179 | 166.4 | 12.62 |
| 45 | LCA_ta | 0.063 | 0.062 | 0.00 | 178 | 165.8 | 12.20 |
| 46 | T23_ta | 0.061 | 0.064 | 0.00 | 180 | 166.5 | 13.51 |
| 47 | LCM_ta | 0.062 | 0.063 | 0.00 | 180 | 166.1 | 13.93 |
| 48 | B_ta | 0.075 | 0.065 | 0.01 | 192 | 175.3 | 16.66 |
| 49 | R_ta | 0.063 | 0.070 | 0.01 | 185 | 169.9 | 15.08 |
| 50 | Kiwi_ta | 0.067 | 0.077 | 0.01 | 180 | 172.7 | 7.29 |
| 51 | S_ta | 0.058 | 0.074 | 0.02 | 197 | 182.4 | 14.56 |
| 52 | NSFE1_ta | 0.056 | 0.079 | 0.02 | 190 | 180.2 | 9.77 |
| 53 | Q_ta | 0.059 | 0.079 | 0.02 | 186 | 184.6 | 1.42 |
| 54 | NSFE2_ta | 0.059 | 0.077 | 0.02 | 188 | 186.5 | 1.52 |
| 55 | NSFE4_ta | 0.065 | 0.079 | 0.01 | 185 | 189.0 | 4.03 |
| 56 | NSFE5_ta | 0.065 | 0.077 | 0.01 | 183 | 189.6 | 6.57 |
| 57 | P_ta | 0.059 | 0.074 | 0.01 | 185 | 189.0 | 3.97 |
| 58 | BBA_ta | 0.049 | 0.083 | 0.03 | 204 | 194.9 | 9.09 |
| 59 | Menemsha | 0.06 | 0.079 | 0.02 | 195 | 185.6 | 9.39 |
| 60 | NES763_ta | 0.069 | 0.058 | 0.01 | 181 | 185.0 | 4.01 |
| 61 | CXL_ta | 0.051 | 0.072 | 0.02 | 183 | 194.0 | 11.01 |
| 62 | Picket | 0.053 | 0.056 | 0.00 | 182 | 189.7 | 7.71 |

**References**

Andersen, O. (2008) The DTU10 global Mean sea surface and Bathymetry. In: *Presented at EGU2008*. Vienna.

Cheng, Y. and Andersen, O.B. (2010) Improvement in global ocean tide model in shallow water regions. Poster, SV. In: *Ostst*. Lisbon, pp 1–68.

Johns, B. ed (1983) *Physical Oceanography of Coastal and Shelf Seas*, 35th edn. Elsevier, Oxford.

Moody, J.A., Butman, B., Beardsley, R.C., et al. (1984) Atlas of tidal elevation and current observation on the Northeast American Continental Shelf and Slope. Alexandria VA.

Ray, R.D. (1999) *A Global Ocean Tide Model From TOPEX/POSEIDON Altimetry: GOT99.2*.

SAIC (2005) Gulf of Maine Mapping Initiative , Priority 1 Area Survey Report (Doc. 05-TR-017). Rhode Island.

USGS (2013) 3 arc second digital elevation model of the Gulf of Maine: Open-File Report 2011-1127. Woods Hole.

Saha, S., et al. 2010. *NCEP Climate Forecast System Reanalysis (CFSR) Selected Hourly Time-Series Products, January 1979 to December 2010*. Research Data Archive at the National Center for Atmospheric Research, Computational and Information Systems Laboratory. https://doi.org/10.5065/D6513W89. Accessed 21/07/2015.

Saha, S., et al. 2011, updated monthly. *NCEP Climate Forecast System Version 2 (CFSv2) Selected Hourly Time-Series Products*. Research Data Archive at the National Center for Atmospheric Research, Computational and Information Systems Laboratory. https://doi.org/10.5065/D6N877VB. Accessed 21/07/2015.
