# Supplementary material for: Dynamic occupancy modeling of temperate marine fish in area‐based closures
Source: Ecol Evol. 2018 Sep 21;8(20):10192–205. doi: 10.1002/ece3.4493 (PMC6206187; doi:10.1002/ece3.4493)
Supplement: Supplementary file 2 [file ECE3-8-10192-s002.docx]

model {

# Specify prior distributions for model parameters

for(i in 1:5){

beta[i] ~ dt(0, pow(1.566267,-2), 7.63179)

}

psi1 ~ dunif(0,1)

for(i in 1:(nyear-1)){

gamma[i] ~ dunif(0,1)

phi[i]~ dunif(0,1)

}

# Specify the state model

for(i in 1:nsite){

z[i,1] ~ dbern(psi1)

for(t in 2:nyear){

muZ[i,t]<- z[i,t-1]*phi[t-1] + (1-z[i,t-1])*gamma[t-1]

z[i,t] ~ dbern(muZ[i,t])

}

}

# Observation model conditional on occupancy state

for (t in 1:nyear){

for(i in 1:nsite){

for(j in 1:nrep) {

D1[i,j,t] <- equals(al[i,j,t], 1) # create dummy variables for factors (algae)

D2[i,j,t] <- equals(al[i,j,t], 2)

cur[i,j,t] ~ dunif(min_cur, max_cur) # impute missing current values

eff[i,j,t] ~ dunif(min_eff, max_eff) # impute missing effort values

logit.p[i,j,t] <- beta[1] + # intercept when algae = 1

beta[2] * cur[i,j,t] +

beta[3] * eff[i,j,t] +

beta[4] * D2[i,j,t] +

beta[5] * D2[i,j,t] * cur[i,j,t]

p[i,j,t] <- exp(logit.p[i,j,t]) / (1 + exp(logit.p[i,j,t]))

y[i,j,t] ~ dbern(eff.p[i,j,t])

eff.p[i,j,t] <- p[i,j,t]*z[i,t]

# Calculate Pearson residuals and generate new data for Bayesian p-values

Var.y[i,j,t] <- sqrt(p[i,j,t] * (1 - p[i,j,t])) + 1E-15 # add small constant to avoid dividing by zero

PearsonResid[i,j,t] <- (y[i,j,t] - p[i,j,t]) / Var.y[i,j,t] # calculate Pearson residuals

y.new[i,j,t] ~ dbern(eff.p[i,j,t]) # estimate new data

PearsonResid.new[i,j,t] <- (y.new[i,j,t]-p[i,j,t]) / Var.y[i,j,t] # calculate Pearson residuals new data

}

}

}

# Calculate fit for Bayesian p-values

fit <- sum(PearsonResid[,,])# Discrepancy for actual data set

fit.new <- sum(PearsonResid.new[,,]) # Discrepancy for replicate data set

# Compute sample and population occupancy, growth rate and turnover

psivec[1]<-psi1 # number of sites occupied in year 1

psi.fs[1]<-sum(z[,1])/nsite # finite sample estimate in year 1

for(t in 2:nyear){

psivec[t] <- psivec[t-1]*phi[t-1] + (1-psivec[t-1])*gamma[t-1] # number of sites occupied in year t

psi.fs[t] <- sum(z[,t])/nsite # finite sample estimate in year t

growthr[t-1] <- psivec[t]/psivec[t-1] # growth rate

turnover[t-1] <- ( 1 - psivec[t-1]) * gamma[t-1]/psivec[t] # turnover rate

}

}
